# Single-cell analysis of multiple myelomas refines the molecular features of bortezomib treatment responsiveness

**DOI:** 10.1038/s12276-022-00884-z

**Published:** 2022-11-15

**Authors:** Seung-Hyun Jung, Sung-Soo Park, Ji-Young Lim, Seon Yong Sohn, Na Yung Kim, Dokyeong Kim, Sug Hyung Lee, Yeun-Jun Chung, Chang-Ki Min

**Affiliations:** 1 Department of Biochemistry, College of Medicine, The Catholic University of Korea, Seoul, South Korea; 2 Department of Biomedicine & Health Sciences, College of Medicine, The Catholic University of Korea, Seoul, South Korea; 3 Department of Hematology, Seoul St. Mary’s Hematology Hospital, Seoul, South Korea; 4 Leukemia Research Institute, College of Medicine, The Catholic University of Korea, Seoul, South Korea; 5 Precision Medicine Research Center/IRCGP, College of Medicine, The Catholic University of Korea, Seoul, South Korea; 6 Cancer Evolution Research Center, College of Medicine, The Catholic University of Korea, Seoul, South Korea; 7 Department of Pathology, College of Medicine, The Catholic University of Korea, Seoul, South Korea; 8 Department of Microbiology, College of Medicine, The Catholic University of Korea, Seoul, South Korea

**Keywords:** Myeloma, Cancer microenvironment

## Abstract

Both the tumor and tumor microenvironment (TME) are crucial for pathogenesis and chemotherapy resistance in multiple myeloma (MM). Bortezomib, commonly used for MM treatment, works on both MM and TME cells, but innate and acquired resistance easily develop. By single-cell RNA sequencing (scRNA-seq), we investigated bone marrow aspirates of 18 treatment-naïve MM patients who later received bortezomib-based treatments. Twelve plasma and TME cell types and their subsets were identified. Suboptimal responders (SORs) to bortezomib exhibited higher copy number alteration burdens than optimal responders (ORs). Forty-four differentially expressed genes for SORs based on scRNA-seq data were further analyzed in an independent cohort of 90 treatment-naïve MMs, where 24 genes were validated. A combined model of three clinical variables (older age, low absolute lymphocyte count, and no autologous stem cell transplantation) and 24 genes was associated with bortezomib responsiveness and poor prognosis. In T cells, cytotoxic memory, proliferating, and dysfunctional subsets were significantly enriched in SORs. Moreover, we identified three monocyte subsets associated with bortezomib responsiveness and an MM-specific NK cell trajectory that ended with an MM-specific subset. scRNA-seq predicted the interaction of the *GAS6*-*MERTK, ALCAM*-*CD6*, and *BAG6-NCR* gene networks. Of note, tumor cells from ORs and SORs were the most prominent sources of *ALCAM* on effector T cells and *BAG6* on NK cells, respectively. Our results indicate that the complicated compositional and molecular changes of both tumor and immune cells in the bone marrow (BM) milieu are important in the development and acquisition of resistance to bortezomib-based treatment of MM.

## Blood cancer: Molecular basis for treatment resistance explored

Single-cell RNA sequencing reveals insights into treatment resistance in multiple myeloma, a form of blood cancer, and provides possible molecular targets for personalised therapies. Disease progression depends on the interactions between tumor cells and tumor microenvironments in the bone marrow. Bortezomib is used to treat myeloma, but success is hampered by poor responses and resistance in certain patients. To identify potential biomarkers for bortezomib resistance, Seung-Hyun Jung at The Catholic University of Korea, Seoul, South Korea, and coworkers conducted single-cell RNA sequencing on bone marrow aspirate samples from 18 patients with myeloma. They identified 24 genes overexpressed in poor responders, and three further clinical variables associated with poor prognosis. Certain groups of T cells were also enriched in poor responders, suggesting that complex communications between tumor and immune cells may underpin resistance.

## Introduction

Multiple myeloma (MM) is a hematologic malignancy characterized by clonal plasma cell proliferation^1^. Advances in novel strategies have provided breakthroughs in treatments for MM patients over the last decades^2,3^. These advances involve not only the use of various classes, including proteasome inhibitors, immune modulators, monoclonal antibodies, or B-cell-mutated antigens but also the introduction of various generations of each class^2–4^. Nevertheless, MM remains incurable because innate and acquired resistance may inevitably develop during treatment. Therefore, there is a continuing need to develop personalized treatments with optimal therapeutic options for MM.

Due to the limitation of direct comparative trials between novel agent therapies for the selection of optimal therapy, a biomarker-driven personalized selection of therapies for MM has been regarded as a realistic alternative^5^. Bortezomib is an anticancer medication used to treat MM and mantle cell lymphoma. Blocking targeted proteolysis, normally performed by the 26S proteasome, prevents the degradation of proapoptotic factors, thereby triggering programmed cell death in tumor cells^6^. This molecule is widely used as a key drug for induction, consolidation, maintenance, or salvage therapy for MM in combination with other agents^7^. The overall response rate of newly diagnosed MM to bortezomib and dexamethasone is ~67%, but that of relapsed refractory MM is reduced to 40–60%^8^. Thus, the identification of molecular markers predicting the response to bortezomib-based therapy is valuable. However, to date, there is no definite way to determine patient response to bortezomib-based treatment^9^.

Mounting evidence has demonstrated that the tumor microenvironment (TME) plays a crucial role in cancer pathogenesis^10,11^ and patient response to treatment^12,13^. For example, distinct TMEs existing within a cancer tissue may dictate the heterogeneous fates of tumor lesions following cancer therapies^10^. TME signatures effectively predict responses to chemotherapy in gastric cancer patients^13^. Malignant plasma cell dependence on the bone marrow (BM) TME is the main feature of MM^14^. Interestingly, bortezomib induces an anti-MM immune response, and an immune cell death-related signature predicts clinical outcomes in MM patients after bortezomib treatment^15^. Thus, a comprehensive understanding of both MM and the TME will enable the discovery of predictive biomarkers and improve personalized treatments. The BM TME of MM patients comprises many cell types; thus, individual cell identification is important for the analysis. Conventional “bulk” RNA-sequencing methods process a mixture of all cells, averaging out underlying differences in cell-type-specific transcriptomes^16^. In contrast, single-cell RNA-sequencing (scRNA-seq) profiles the gene expression of individual cells and decodes the intercellular signaling networks, allowing the identification of individual cellular states in many tumors^16^. scRNA-seq also provides more precise insights into the TME, such as a mechanism representing intratumor and interpatient heterogeneities, as well as cell–cell interactions through ligand–receptor signaling^17,18^. To date, several studies using scRNA-seq have analyzed the BM tissues of MM patients to identify the expression of individual cells in either tumor or TME cells^19–23^. However, scRNA-seq signatures for bortezomib treatment responsiveness have not been identified in the TME of MMs, and the identification of the cell–cell interactions between the tumor and TME is still unclear in MMs.

In this study, we performed scRNA-seq analyses of BM tissues from MM patients receiving bortezomib-based treatments to identify the following: (i) single-cell-level differences in BM cells between optimal responders (ORs) and suboptimal responders (SORs) to bortezomib and (ii) the genes and cell–cell communication networks associated with treatment responsiveness.

## Materials and methods

### Tumor specimens

For scRNA-seq, we used baseline BM aspirates from 18 newly diagnosed MM patients who received bortezomib–melphalan–prednisolone treatment. As a validation cohort, we used 90 treatment-naïve BM aspirates from 50 bortezomib–melphalan–prednisolone-treated and 40 bortezomib–thalidomide–dexamethasone-treated MM patients. The Clinical Outcomes in Multiple Myeloma to Personal Assessment of Genetic Profile (CoMMpass) dataset was also used as an independent validation cohort (https://research.themmrf.org/). Detailed information for patients and sample preparation is available in the Supplementary Methods. The study design and overall strategy are illustrated in Supplementary Fig. 1. This study was approved by the Institutional Review Board of the Catholic University of Korea, College of Medicine (approval number: KC12SISE0594).

### Single-cell RNA sequencing

The single-cell library was prepared using a commercially available droplet method, the Chromium System from 10× Genomics, Inc. (Pleasanton, CA, USA), and a Single Cell 3′ v3 Reagent Kit according to the manufacturer’s protocol. Sequencing reads were mapped to the GRCh38 reference genome, and bioinformatics processing of the scRNA-seq data was performed using R packages. The detailed methods, including scRNA-seq, bioinformatic analyses, copy number alteration (CNA) detection, and cell–cell communication analysis, are available in the Supplementary Methods.

### TaqMan low-density gene expression array experiments

For validation of the treatment response-related genes using the validation cohort, 44 differentially expressed genes (DEGs) (upregulated: *BCAP31*, *BCL2*, *BST2*, *CCL3*, *CCND1*, *CD320*, *CD53*, *COX5A*, *CXCR4*, *DUSP2*, *EEF1B2*, *EGR1*, *EIF2AK4*, *EIF3M*, *HIST1H1C*, *HSP90AB1*, *IL6ST*, *JUNB*, *LAMP5*, *MS4A1*, *MYC*, *NFKBIA*, *NOP53*, *NPM1*, *PDIA2*, *PIM1*, *PSMA7*, *RACK1*, *RGS1*, *SEC11A*, *SQSTM1*, *SRP9*, *SSR3*, *TNFRSF17*, *TSC22D3*, and *UQCRH*; downregulated: *ATF5*, *IGF1*, *ITGB7*, *NEB*, *NSD2*, *PPP1R10*, *TIMP2*, and *RHOB*) between the optimal and suboptimal responder groups were examined using custom-made TaqMan low-density arrays (TLDA). The expression level of each target gene was calculated using 2^−ΔΔCt^, where ΔCt is the difference in threshold cycles for the sample in question normalized against the endogenous control gene (18S ribosomal RNA). The detailed methods for the TLDA validation are available in the Supplementary Methods.

### Statistical analysis

Fisher’s exact test was used for categorical variables. Student’s *t*-test was used for continuous variables. The relationships between the proportions of the cell types and treatment responses were evaluated using Spearman’s rank correlation. Linear discriminant analysis was applied to construct a prediction model for bortezomib-based treatment response. The receiver operating characteristic (ROC) curve and area under the curve (AUC) was used to assess the predictive values of each DEG and the prediction model for bortezomib-based treatment. For survival analysis, time-to-event variables were defined as the duration from the initiation date of bortezomib-based treatment to the date of disease progression. Patient survival was calculated by the Kaplan–Meier method, and differences in survival rates between groups were tested with the log-rank test. Statistical analyses were performed using SPSS (version 25, Chicago, IL). GraphPad Prism software (version 8, La Jolla, CA) was used to create graphs. All *P* values < 0.05 were considered significant in all statistical analyses.

## Results

### Single-cell sequencing identified 12 types of plasma cells and microenvironment cells in MMs

**Fig. 1:**
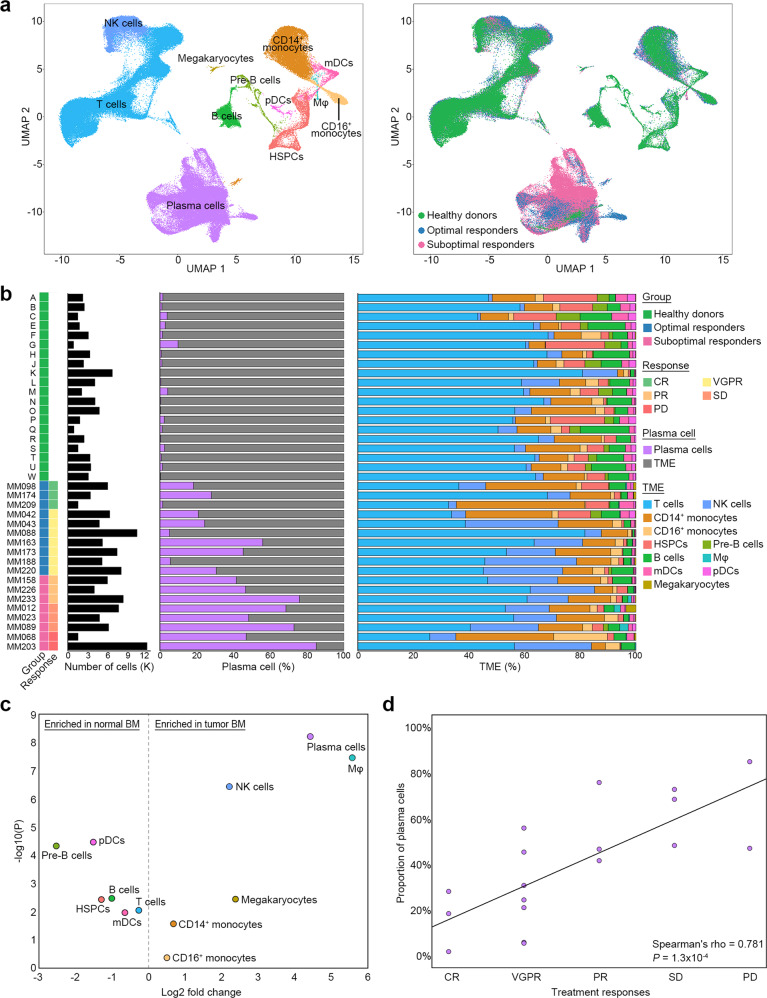
Tumor and immune landscapes of BMs in healthy donors and MM patients. **a** The scRNA-seq data of BM aspirates from 20 healthy donors and 18 MM patients were integrated. Left: two-dimensional uniform manifold approximation and projection (UMAP) visualization of 164,521 BM MNCs identified 12-cell types after unsupervised clustering. Each point represents a single cell and is colored based on cell types. mDCs myeloid dendritic cells, Mφ macrophages, HSPCs hematopoietic stem, and progenitor cells, pDC plasmacytoid dendritic cells. Right: UMAP plot colored by clinical groups. **b** Cell type composition distribution for each sample. Samples are ordered according to the groups and treatment responses. TME tumor microenvironment, CR complete response, VGPR very good partial response, PR partial response, SD stable disease, PD progressive disease. **c** Immune composition changes between the normal and MM samples. For each cell type, the log fold change in the mean cell fraction between the MM and normal samples, with log-transformed *P* values on the *y*-axis, is shown. **d** The proportion of plasma cells correlated with treatment responses from CR to PD. A correlation was assessed by the Spearman coefficient with 18 MM patients.

We performed scRNA-seq of BM-mononuclear cells (MNCs) isolated from 18 treatment-naïve MM patients (Supplementary Tables 1 and 2) who later received bortezomib-based treatments and were divided into two groups according to treatment responsiveness: optimal and suboptimal (Supplementary Methods). Publicly available scRNA-seq data of BM-MNCs from 20 healthy donors^24^ were used as normal control. After quality control, we obtained 164,521 BM-MNCs from MM patients (ORs [*n* = 10]: 58,282 cells; SORs [*n* = 8]: 50,373 cells) and healthy donors (55,866 cells). Unsupervised clustering identified 12 cell populations: plasma cells, T cells, NK cells, CD14^+^ monocytes, CD16^+^ monocytes, hematopoietic stem and progenitor cells, pre-B cells, B cells, macrophages (Mφs), myeloid dendritic cells, plasmacytoid dendritic cells, and megakaryocytes (Fig. 1a; Supplementary Fig. 2 and Supplementary Table 3). The proportion of plasma cells was significantly higher in the MM group than in the control group (43.9% versus 1.2% of total BM-MNCs, *P* = 5.8 × 10^−9^, Fig. 1b and c). Notably, a significant relationship was found between plasma cell proportions and treatment responses (Spearman’s rho = 0.781, *P* = 1.3 × 10^−4^, Fig. 1d). Mφ, NK cell, megakaryocyte, and CD14^+^ monocyte populations were significantly enriched in the MM group compared to the control group, whereas plasmacytoid dendritic cells, pre-B cell, B cell, hematopoietic stem and progenitor cell, T cell, and myeloid dendritic cell populations were diminished (Fig. 1b and c)^21^.

### Heterogeneity of multiple myeloma cells

**Fig. 2:**
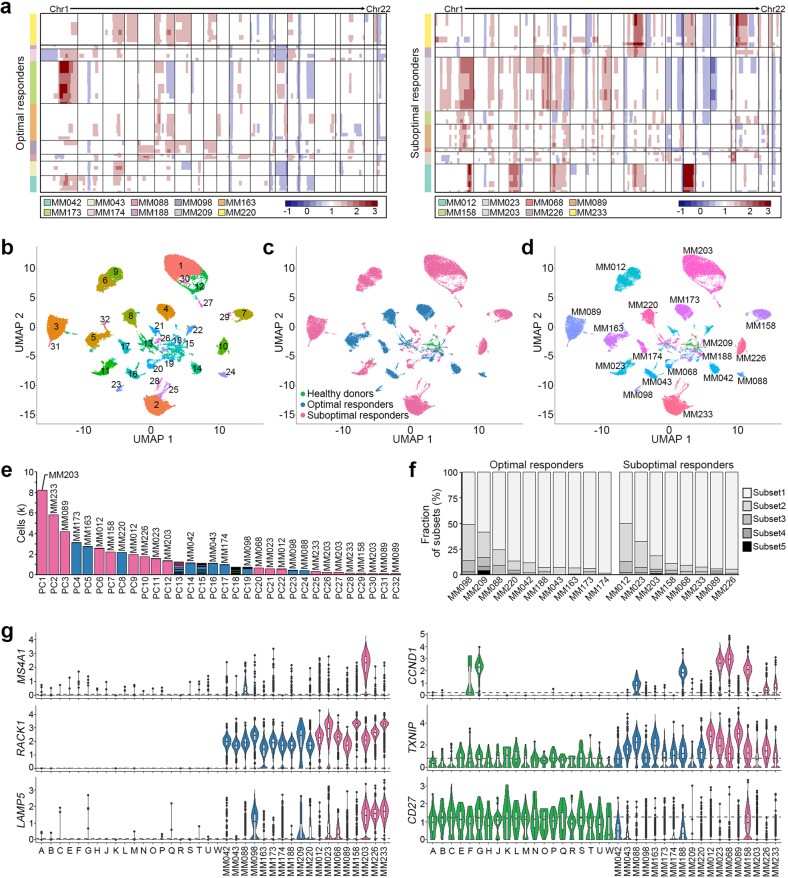
Plasma cell subsets of MM in scRNA-seq. **a** Genome-wide heatmap displaying CNA profiles of plasma cells from 18 MM patients (left: ORs; right: SORs). CNAs were inferred from the scRNA-seq data using the inferCNV R package. Red and blue indicate copy number gains and losses, respectively. The *X*-axis shows chromosomes in numerical order. Plasma cells from different MM patients are indicated as different color bars on the bottom of the heatmap. **b**–**d** UMAP plot colored based on plasma cell subsets (**b**), clinical groups (**c**), and patients (**d**). **e** Bar plot representing the number of cells per plasma cell subset. Each bar is color-coded according to clinical groups as shown in (**c**). The names above the bars correspond to the individual cases, with the majority of cells in each cluster. **f** Bar plot representing plasma cell composition in individual ORs and SORs. **g** Violin plots present the expression of common MM driver genes in 20 healthy donors (alphabets “A” to “W”) and 18 MM patients (numbers “MM042” to “MM233”). Each violin plot is color-coded according to the clinical groups as shown in (**c**). The gray dotted line represents the average expression in healthy donors.

To distinguish MM cells from normal plasma cells, we examined CNAs using scRNA-seq data. ~10.2% of the plasma cells were considered normal cells without CNAs. Consistent with previous reports, the inferred CNA profiles of the 18 MM samples showed interpatient and intratumor heterogeneities (Fig. 2a)^19,22^. In addition, chromosomal abnormalities detected by karyotyping or fluorescence in situ hybridization were largely compatible with CNAs inferred by scRNA-seq (71%, 52 of 73 abnormality events). The top recurrent CNA was the copy loss on 16p13.3 (17/18), followed by the copy gains on 3q21.3-q27.1 (16/18) and 6q12-q23.3 (15/18; Supplementary Table 4). Of note, SORs harbored higher CNA numbers (median of 37 vs. 22 CNAs, *P* = 8.4 × 10^−4^) and longer CNA lengths (average of 1049.1 vs. 683.0 Mb, *P* = 0.013) than ORs.

scRNA-seq revealed 32 plasma cell subsets (PC1–PC32; Fig. 2b–e; Supplementary Table 5). The plasma cells from healthy donors were grouped into one subset (PC18; Fig. 2b and c). The malignant cell subsets, however, were clustered primarily by patients, and there were multiple subsets (2–5 subsets) in each MM (Fig. 2d–f). The PC13 and PC15 subsets contained T/NK gene signatures that were shared by multiple MM patients, suggesting that they might represent MM-related T/NK-like plasma cell subsets (Fig. 2e; Supplementary Table 5). The representative cases of intratumor heterogeneity are presented in Supplementary Fig. 3. *MS4A1* (CD20), a B-cell marker normally lost in terminally differentiated plasmablasts and plasma cells^25^, was highly expressed in MM203 but not in the other MMs (Fig. 2g; Supplementary Fig. 3), suggesting plasma cell dedifferentiation in this case. Dedifferentiation was morphologically confirmed in the BM aspirate (Supplementary Fig. 4). Well-known driver genes were commonly overexpressed (e.g., *CCND1, RACK1*, *TXNIP*, and *LAMP5*) or underexpressed (e.g., *CD27*) in malignant cells (Fig. 2g; Supplementary Table 6).

### Gene sets associated with bortezomib treatment responses in multiple myeloma

We performed a pseudobulk DEG analysis between the OR (*n* = 10) and SOR (*n* = 8) groups, identifying 1443 genes as SOR-related DEGs (1346 upregulated and 97 downregulated; Supplementary Fig. 5a and Supplementary Table 7). In addition to the MM driver genes (e.g., *CCND1*, *LAMP5*, and *TNFRSF17*)^19^ and quadruple therapy resistance genes (e.g., *NPM1* and *MYC*)^22^, we discovered upregulated DEGs in SORs (e.g., *MS4A1*, *RACK1*, *UQCRH*, and *SQSTM1*). Downregulated DEGs included previously known *NSD2* and *ITGB7* and newly identified DEGs (e.g., *PPP1R10* and *NEB*). The signatures of “ribosome,” “protein folding,” and “proteasome” were enriched in SORs, whereas those of “B-cell-mediated immunity” and “MHC protein complex” were enriched in ORs (Supplementary Fig. 5b).

**Fig. 3:**
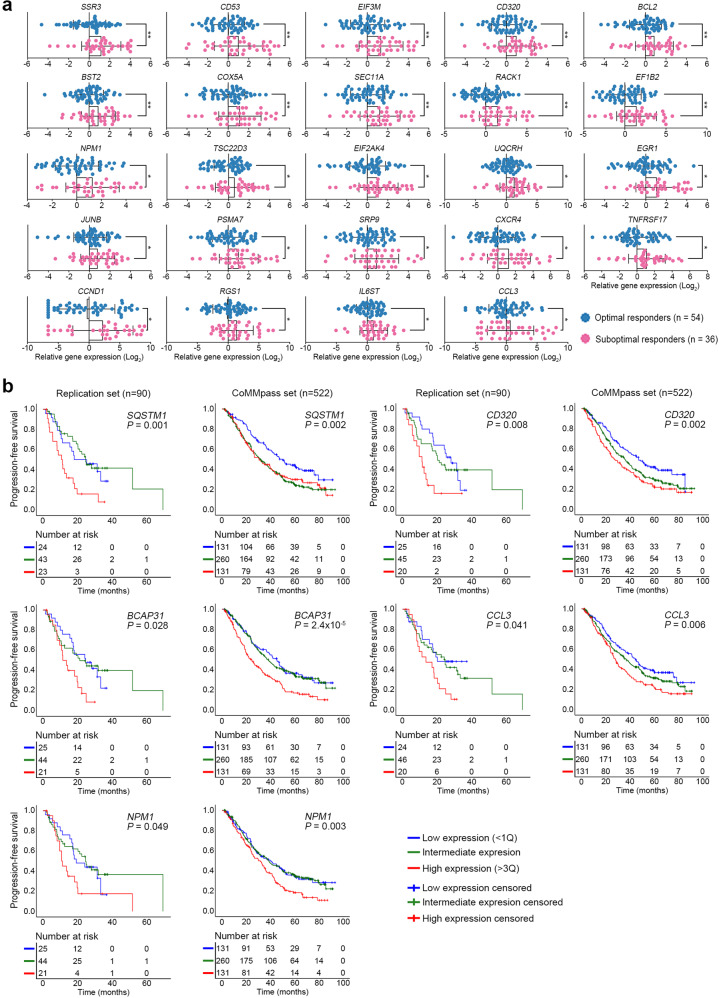
Validation of DEGs associated with bortezomib-based treatment responses. **a** Dot plots represent the expression levels of 24 genes measured by TLDA. The relative gene expression level was normalized against the endogenous control gene (18S rRNA). Statistical significance was calculated between the OR (*n* = 54) and SOR (*n* = 36) groups. The *X*-axis represents the relative gene expression level (log_2_ fold changes) based on the mean of the ORs as the calibrator. The detailed results of the 44 selected genes are presented in Supplementary Table 8. *P value < 0.05; **P value < 0.01. **b** Kaplan–Meier curves for PFS according to the gene expression level. The five genes significantly associated with poor PFS and overall survival in the CoMMpass dataset (*SQSTM1*, *CD320*, *BCAP31*, *CCL3*, and *NPM1*) are illustrated. Patients with higher expression (red, >quartile 3) of each gene showed significantly poorer PFS than those with intermediate (green, quartiles 2 and 3) or lower expression (blue, <quartile 1). The detailed data of the 44 selected genes are listed in Supplementary Tables 8 and 9.

**Fig. 4:**
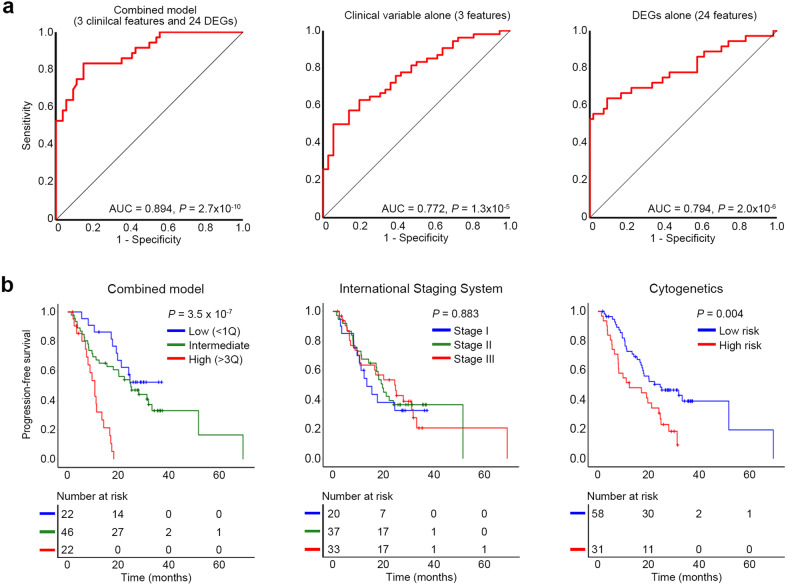
Prediction model for bortezomib-based treatment response. **a** Receiver operating characteristic curves for the combined prediction model, clinical variable alone model, and DEG alone model. AUC: area under the curve. **b** Kaplan‒Meier curves for progression-free survival according to the discriminant score, International Stating System, and cytogenetics. Patients with higher discriminant scores (red, >quartile 3) of the combined prediction model showed significantly poorer progression-free survival than those with intermediate (green, quartiles 2 and 3) or lower discriminant scores (blue, <quartile 1). Regarding cytogenetics, t(4;14), del(17p), and t(14;16) were grouped as high risk.

Based on the significance levels and the previously reported genes strongly related to MM pathogenesis, we selected 44 genes (Supplementary Methods) and analyzed their expression in an independent replication set of 90 treatment-naïve MMs (ORs, *n* = 54; SORs, *n* = 36) using the TLDA assay (Supplementary Table 2). CD138-positive plasma cells isolated by magnetic-activated cell sorting were used for TLDA assays. Twenty-four genes were consistently upregulated in the SORs (Fig. 3a). Of these, 20 were positively correlated with the treatment responses from a complete response to progressive disease (Supplementary Table 8), but none of them were validated in the CoMMpass dataset. We also found another set of 12 genes significantly associated with poor progression-free survival (PFS) (Supplementary Table 8); among these, *SQSTM1*, *CD320*, *BCAP31*, *CCL3*, and *NPM1* were significantly associated with poor PFS and overall survival in the CoMMpass dataset (Fig. 3b; Supplementary Table 9). Among the clinical variables, three (older age, low absolute lymphocyte count, and no autologous stem cell transplantation) were significantly associated with SORs (Supplementary Table 10). Next, we developed a combined prediction model for bortezomib-based treatment response using the three clinical variables and 24 validated DEGs (Supplementary Methods). This prediction model showed a stronger stratification power (AUC = 0.894) than the clinical variables (AUC = 0.772) or DEGs alone (AUC = 0.794; Fig. 4a). The sensitivity, specificity, and accuracy of the combined prediction model were 83.3%, 85.2%, and 84.1%, respectively, based on the discriminant score threshold set at 0.052 (Supplementary Fig. 5c). PFS rates also worsened significantly with increasing discriminant scores, demonstrating superiority to conventional clinical risk stratifications such as the International Staging System (ISS) or cytogenetics (Fig. 4b).

### Molecular features of T-cell subsets in MM bone marrow

**Fig. 5:**
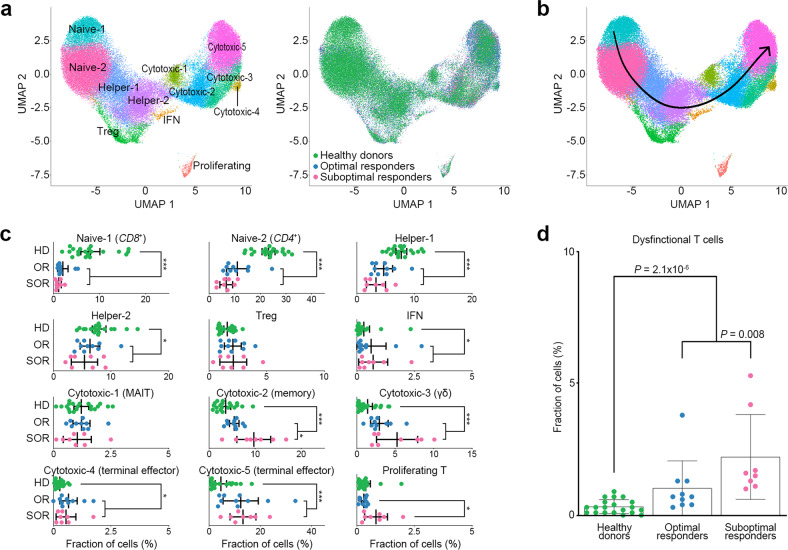
T-cell subsets of MM in scRNA-seq. **a** UMAP plot colored by T-cell subsets (left) and clinical groups (right). **b** Pseudotime trajectory as per the pseudotime algorithm. The trajectory analysis shows a sequential differentiation process of naïve T (naïve-1 and 2) → helper T (helper-1 and 2) → *GZMK*^+^ cytotoxic memory T (cytotoxic-2) → *GZMB*/*GZMH*^+^ terminal effector T (cytotoxic-5). **c** Dot plots represent the proportion of each T-cell subset between healthy donors (*n* = 20), MM ORs (*n* = 10), and MM SORs (*n* = 8). The cytotoxic-2 subset (*CD8B*^+^*GZMK*^+^ memory T population) was significantly enriched in the SORs compared with the ORs (*P* = 0.01). The mean and 95% confidence interval are represented with black lines. *P value < 0.05; **P value < 0.01; ***P value < 0.001; HD healthy donors; ORs optimal responders; SORs suboptimal responders. **d** Dot plots represent the proportion of dysfunctional T cells (dysfunctional module score > 0.5) between healthy donors, MM optimal responders, and MM suboptimal responders.

T cells, the largest TME population (Fig. 1b), comprised 12 subsets of two naïve, two helpers, one regulatory (Treg), one interferon (IFN) signature, one proliferating, and five cytotoxic T cells (Fig. 5a; Supplementary Fig. 6 and Supplementary Table 11). The cytotoxic subsets were *CD4*^*−*^*CD8*^*−*^*GZMK*^+^ mucosal-associated invariant T (cytotoxic-1), *CD8B*^+^*GZMK*^+^ cytotoxic memory (cytotoxic-2), *CD4*^*−*^*CD8*^*−*^*GZMB*^+^*GZMH*^+^ γδ (cytotoxic-3), and *CD4*^+^*/CD8B*^*+*^*GZMB*^+^*GZMH*^+^ terminal effector (cytotoxic-4, 5; Supplementary Fig. 6a and d). Consistent with previous findings^21,26^, the trajectory showed a sequential differentiation process of naïve T cells → helper T cells → *GZMK*^+^ cytotoxic memory T cells → *GZMB*/*GZMH*^+^ terminal effector T cells (Fig. 5b). IFN, cytotoxic memory, γδ, and terminal effector T subsets were significantly enriched in the MM group compared to the control group (Fig. 5c). Of note, cytotoxic memory and proliferating T cells were significantly enriched in the SORs compared to the ORs (*P* = 0.010 and *P* = 0.034, respectively; Fig. 5c). The dysfunctional module score, a distinct gene module for T-cell dysfunction that can be uncoupled from T-cell activation (*PDCD1*, *LAG3*, *HAVCR2*, *TIGIT*, *CD244*, *TOX*, and *CTLA4*)^26^, was higher in cytotoxic (memory, γδ, and terminal effector) and Treg cells than in other subsets (Supplementary Fig. 6c). Dysfunctional T cells (dysfunctional module score > 0.5) were enriched in the MM group compared to the control group (*P* = 2.0 × 10^−6^) as well as in the SORs compared to the ORs (*P* = 0.008; Fig. 5d).

### Molecular features of NK, B, and myeloid cell subsets in MM bone marrow

**Fig. 6:**
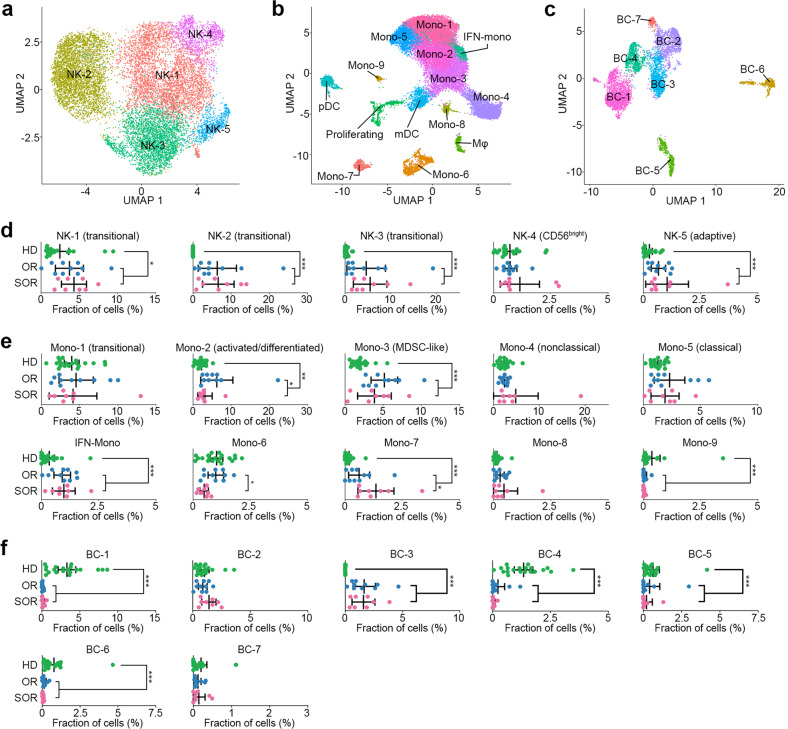
NK, monocyte/DC/Mφ, and B-cell subsets of MM in scRNA-seq. **a** UMAP plot colored by NK cell subsets. **b** UMAP plot colored by monocyte/DC/Mφ cell subsets. **c** UMAP plot colored by B-cell subsets. **d** Dot plots represent the proportion of each NK cell subset between the healthy donors (*n* = 20), MM ORs (*n* = 10), and MM SORs (*n* = 8). **e** Dot plots represent the proportion of each monocyte/DC/Mφ cell subset between the healthy donors (*n* = 20), MM ORs (*n* = 10), and MM SORs (*n* = 8). **f** Dot plots represent the proportion of each B-cell subset between the healthy donors (*n* = 20), MM ORs (*n* = 10), and MM SORs (*n* = 8). *P value < 0.05; **P value < 0.01; ***P value < 0.001; HD healthy donors, OR optimal responders, SOR suboptimal responders.

We identified five NK subsets (Fig. 6a): CD56^bright^ (NK-4 enriched with *GZMK* and *XCL2*), adaptive (NK-5 enriched with *CD3D* and *CD3G*), and three transitional states ranging from “active” to “terminally matured” (NK-1 enriched with *ZFP36* and *FOS*, NK-2 enriched with *JUND* and *ZEB2*, and NK-3 enriched with *S100A4* and *CST7*) (Supplementary Table 11)^27^. These subsets, except NK-4, were higher in the MM group than in the control group (*P* < 0.05); however, their numbers were not significantly different between the ORs and SORs (Fig. 6d). Trajectory analysis revealed a well-established NK cell developmental process (NK4 → NK1 → NK3)^27^, followed by further differentiation into NK-2. This NK2-destined trajectory was evident in MMs but was not detected in healthy donors (Supplementary Fig. 7).

We identified 10 monocyte subsets comprising three groups: *CD14*^+^ (mono-1, 2, 3, 5, and IFN-mono), *FCGR3A*^*+*^ (CD16) nonclassical (mono-4), and the others (mono-6, 7, 8, and 9) (Fig. 6b; Supplementary Table 11). Trajectory analysis revealed a sequential differentiation process: classical monocyte (mono-5) → transitional monocyte (mono-1) → activated monocyte (mono-2; Supplementary Fig. 8). The mono-3 subset resembled myeloid-derived suppressor cells (Supplementary Fig. 8f). Mono-2, mono-3, mono-7, and IFN-mono were significantly enriched in the MM group (Fig. 6e). Mono-2 (*P* = 0.034) and mono-6 (*P* = 0.017) subsets were decreased, whereas mono-7 (*P* = 0.017) was increased in the SORs compared with the ORs (Fig. 6e).

We identified seven B-cell subsets (BC1–BC7); of these, BC-3 was enriched in the MM group compared to the control group (Fig. 6c and f). This subset highly expressed prototypical B-cell markers as well as MM-related genes (e.g., *RACK1*, *NOP53*, and *ATP5MG*; Supplementary Fig. 9). The signatures of “oxidative phosphorylation” and “response to oxidative stress” were enriched in BC-3 (Supplementary Fig. 9).

### Prediction of cell–cell communication between multiple myeloma and TME cells

**Fig. 7:**
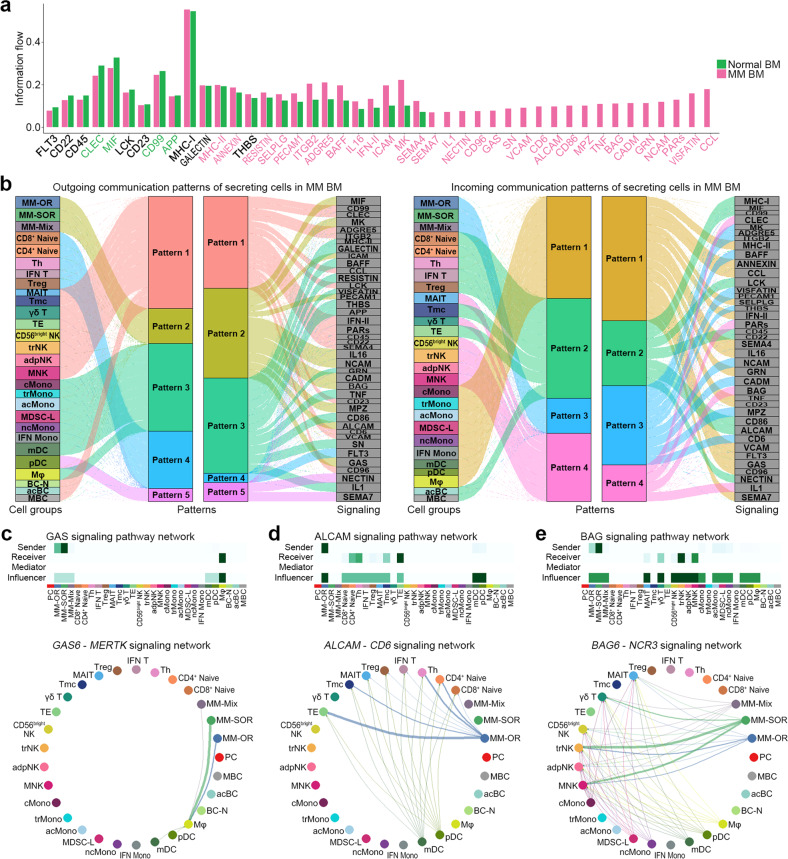
Cell–cell communication analyses of the tumor and TME. **a** Bar plot representing all significant signaling pathways ranked based on their differences in overall information flow within the inferred networks between normal BM and MM BM. The overall information flow of a signaling network was calculated by summarizing all the communication probabilities in the network. The *X*-axis represents all significant signaling pathways. Signaling pathways marked in pink are more enriched in MM BM, whereas those marked green are more enriched in normal BM. **b** Left: the inferred outgoing communication patterns of secreting cells visualized by the alluvial plot, which shows the correspondence between the inferred latent patterns and cell groups as well as signaling pathways. The thickness of the flow indicates the contribution of the cell group or signaling pathway to each latent pattern. The height of each pattern is proportional to the number of its associated cell groups or signaling pathways. Right: the inferred incoming communication patterns of target cells. Incoming patterns show how the target cells coordinate with each other and with certain signaling pathways to respond to incoming signaling. **c**–**e** Top, the heatmap shows the relative importance in each cell group based on the computed network centrality measures of the GAS (**c**), ALCAM (**d**), and BAG (**e**) signaling networks. The heatmap scale indicates importance, from white (importance = 0) to dark green (importance = 1). Bottom, circle plots showing inferred autocrine and paracrine signaling to MM BM cell subsets and to normal BM cell subsets, respectively. Edge width represents the communication probability. Edge colors are consistent with the signaling source. MM-OR multiple myeloma cells from ORs, MM-SOR multiple myeloma cells from SORs, MM-Mix multiple myeloma cells from both ORs and SORs, IFN T interferon signature T cell, Tmc cytotoxic memory T cell, TE terminal effector T cell; trNK transitional NK cell, adpNK adaptive NK cell, MNK NK cell from MM (NK-2 subset), acMono activated monocyte, MDSC-L MDSC-like monocyte, IFN Mono interferon signature monocyte, acBC activated B cell, MBC B-cell from MM (BC-3 subset), PC plasma cell from healthy donors, Th helper T cell, trMono transitional monocyte, ncMono nonclassical monocyte, BC-N B-cell from healthy donors (BC-1 and BC-4 subsets).

To explore the potential interactions between tumor cells and the TME in MM, we examined ligand‒receptor pairs among the cell subsets^28^. The number of inferred interactions was higher in the MM group than in the control group (Supplementary Fig. 10a and Supplementary Table 12). Based on topological similarity, the interactions were divided into four signaling groups (Supplementary Fig. 10b). Of the 44 putative interactions detected, 32 were enriched in the MM group (Fig. 7a, in pink letters), whereas 4 were enriched in the control group (Fig. 7a, in green letters). Of note, 19 interactions were exclusively identified in the MM group (Fig. 7a, single pink bars).

Communication pattern analysis identified five outgoing and four incoming paths (Fig. 7b). The outgoing pattern shows how cells as signal sources modulate certain signaling pathways to drive communication, while the incoming pattern shows how cells as signal receivers modulate certain signaling pathways to respond to incoming signals^28^. The largest pattern of outgoing MM signaling was pattern #2, which included NCAM, CADM, BAG, MPZ, ALCAM, and GAS (Fig. 7b). Of these, CADM, MPZ, and NCAM simultaneously appeared in the incoming signaling (incoming pattern #3), suggesting autocrine or homophilic interactions in MM (Fig. 7b; Supplementary Fig. 10c). *GAS6* from MM was predicted to interact with Mφ along the *GAS6*-*MERTK* axis (Fig. 7c). Of note, the MMs from ORs (MM-OR) were the most prominent sources of CD6 ligand (*ALCAM*) acting on effector T cells, whereas those from the SORs (MM-SOR) were the most prominent sources of the NCR ligand (*BAG6*) acting on activated NK cells (Fig. 7d and e). Another interesting pathway was the CCL interaction, where the SOR’s cytotoxic T cells, NK cells, and MMs were the predominant sources of *CCR1* ligand potentially acting on monocyte populations (activated monocyte and MDSC-like; Supplementary Fig. 10d). Of note, the *CCL5*-*CCR1* pair was highly enriched between T/NK cells and monocytes, whereas the *CCL3*-*CCR1* pair was highly enriched between MMs and monocytes, suggesting context-dependent interactions of *CCR1* in MM (Supplementary Fig. 10d).

## Discussion

The main aim of our study was twofold: the first was to find scRNA-seq-based altered expression in MM tissues for the SORs to bortezomib treatments, and the second was to find the cellular subsets and cell–cell communication networks associated with bortezomib responsiveness. Our data suggest three major conclusions: SORs and poor survival in MM can be predicted based on the gene sets of MM cells; second, in the TME, cytotoxic memory T, dysfunctional T, proliferating T, and mono-7 subsets are significantly enriched in the SORs compared to the ORs; and third, MMs potentially communicate with the immune cells through several ligand‒receptor pairs, some of which are associated with either the ORs or SORs. Altogether, these scRNA-seq-based findings regarding bortezomib responsiveness provide a rich resource for exploring the molecular targets and underlying resistance mechanisms in MM.

Notably, the SORs harbored higher CNA numbers and longer CNA lengths than the ORs, suggesting that chromosomal instability causes bortezomib unresponsiveness. One case (MM203) showed a plasma cell dedifferentiation pattern in scRNA-seq, which was confirmed in the BM aspirate. Immunoglobulin synthesis and secretion activity are gradually enhanced during plasma cell maturation, thus suggesting that the differentiation degree of MMs may impact bortezomib sensitivity^29^. Plasma cell dedifferentiation is a therapeutic escape mechanism that allows tumor cells to develop resistance to proteasome inhibitors^30^, but the resistance can be reversed by inducing the expression of plasma cell maturation markers^29,31^. Consistent with these findings, MM203 is an SOR with MM that is refractory to bortezomib therapy.

The scRNA-seq DEGs combined with TLDA validation identified 24 genes significantly upregulated in the SORs, including *CCND1, JUNB, BCL2, CD53, CD320*, and *RACK1*. *CCND1* is overexpressed in t(11;14) MM by rearrangement and in the majority of hyperdiploid MM by other mechanisms; however, prognostic or treatment predictive values of t(11;14) remain uncertain^32^. Our data are also consistent with previous reports of the poor survival of MM patients with *CCND1* amplification^33^. *CD53* is important in plasmablastic differentiation in MM, and its abnormal expression is reported in B-cell chronic lymphocytic leukemia^34^. *JUNB* expression is essential for dexamethasone and bortezomib resistance in a mouse MM model^35^. *CCL3* expression is involved in osteolytic lesion formation in MM^36^. *RACK1*, which plays important roles in cell migration, invasion, and chemotherapy resistance, is associated with bortezomib resistance in MM cells^37^. *BCL2* is an antiapoptotic protein that is associated with clinically aggressive behavior and suboptimal response to therapies in MM cells and other B-cell malignancies^38^, which is in agreement with our data. However, data on the association between *BCL2* expression and bortezomib responses are conflicting and suggest that *BCL2* is not a monolithic factor for determining drug responses. Many other genes, such as *TSC22D3, EIF2AK4, PSMA7*, and *SRP9*, are not known for their association with MM or bortezomib resistance. The 24 overexpressed genes associated with poor bortezomib responses perform different biological functions, indicating that there may be many alterations in expression underlying bortezomib unresponsiveness and suggesting that a gene set rather than one or two gene applications would be required to precisely predict the responses.

Several gene signatures have predicted the overall prognosis and response to individual therapies in MM^9,39^, including the seven-gene signature for bortezomib responsiveness in the PADIMAC study^40^. Moreover, clinical features, such as the ISS, hyperdiploidy, high-risk cytogenetics, and peripheral blood cell counts, are being used widely to predict treatment responsiveness. However, the predictive power of the gene signatures or the clinical features is not sufficient for clinical application due to inconsistency^39^. Likewise, no single gene (AUC = 0.530–0.699) or clinical factor (AUC = 0.514–0.699) was adequate for clinical application in our cohort. Rather, the combined prediction model with three clinical variables (older age, low absolute lymphocyte count, and no autologous stem cell transplantation) and the 24 genes showed highly improved prediction for both bortezomib responsiveness and PFS, suggesting its clinical usefulness. Provided that the CoMMpass dataset differs in treatment regimen and ethnicity, our prediction model should be further investigated in a larger cohort with the same clinical setting.

Bortezomib and other immunomodulatory drugs have anti-MM effects on tumor and TME cells^15^, indicating the importance of TME in MM treatment. MM progression precludes the formation of classical memory T cells, resulting in a dysfunctional T state and immunosuppressive BM^26,41^. In MM, however, the frequency of CD8^+^ terminally exhausted T cells is very low^42^, warranting further dissection of T-cell status in MM. In our data, *GZMK*^+^ cytotoxic memory, proliferating, and dysfunctional T subsets were significantly enriched in the SORs. In agreement with our results, the pan-cancer T-cell atlas showed that MM had strong *GZMK*^+^ exhausted CD8^+^ T-cells in the major exhaustion paths^42^. Conversely, an earlier study reported low *GZMK*^+^ cytotoxic memory T cells with no difference in T-cell exhaustion genes in MM^21^. This finding might be explained by recent data showing that there are two fates of CD8^+^ memory T cells, one of which expresses *GZMK* along with *PDCD1* and *TIGIT* (termed T_PEX_)^41^. These studies did not analyze the dysfunctional T state with treatment responses. Taken together, our data suggest that T-cell activation followed by a gradual waning to an exhausted phenotype is involved in both MM development and responsiveness to the treatments. We found four NK cell subsets; these subsets, particularly the MM-specific NK-2 subset that is highly enriched in MM, suggested that there might be transcriptionally unique states in MM. We identified one MM-enriched B-cell subset (BC-3) that highly expressed MM-related genes and OXPHOS pathway genes^43^, suggesting the existence of unique B cells in the MM TME sharing both B-cell and plasma cell transcription features. Of the 10 monocyte subsets, 4 (mono-2, mono-3, mono-7, and IFN-mono) were significantly enriched in MMs. With regard to treatment responses, low mono-2 and mono-6 and high mono-7 were evident in the SORs. Collectively, the complicated compositional changes of immune cells in the BM milieu may be associated with the development of MM and the acquisition of resistance to bortezomib-based treatment.

Given that the treatment regimen in this study was based on bortezomib, which does not rely on immune cells as the most important mode of action, the changes mentioned above might be caused by bortezomib-based treatment effects on other cells that interact with TME cells. Our unsorted scRNA-seq strategy enabled cell–cell communication analysis between the tumor and TME in the BM of MM patients, revealing that the tumor cell populations might be the major source of cancer-related signals involving *NCAM, MPZ, GAS6, ALCAM, BAG*, and *CCL*. *GAS6*, which encodes the GAS ligand, is overexpressed in many cancers and promotes cancer cell proliferation and survival^44^. Previously, *GAS6* was reported to be produced by BM stromal cells in a mouse model^45^, but our study identified MM cells as the source. The inhibition of *GAS6* or its receptor, *MERTK*, reduced tumor burden in an MM model^45^, suggesting clinical availability. *ALCAM*–*CD6* is known for T-cell effector functions through T-cell activation and proliferation^46^, which is consistent with our findings of enriched *ALCAM*–*CD6* interactions in ORs. *BAG6* can be either activating or inhibitory toward NK cells depending on the secreted or exosomal version^47^. Specifically, the *BAG6*–*NCR3* (NKp30) axis is critical for inhibiting the NKp30 receptor-dependent cytotoxicity of NK cells and promotes immune evasion^48^, which supports our observation that *BAG6*–*NCR3* is enriched in the SORs. This result suggests that MM cells can activate or suppress antitumor immunity by communicating with immune cells, which may be linked to bortezomib treatment responsiveness. We also identified *CCR1* interactions in SORs. The *CCL5*–*CCR1* interaction contributes to the recruitment of monocytes into inflamed tissue^49^, whereas the *CCL3*–*CCR1* interaction causes increased osteolysis and promotes MM dissemination^50^, supporting our prediction of the role of CCR1 in MM. Nevertheless, a limitation of our study is that scRNA-seq primarily measured transcript levels without analyzing actual cellular phenotypes. Additionally, the standard treatment for MM has been changed to daratumumab-based treatments. However, the new options still include bortezomib in combinations such as daratumumab–bortezomib–melphalan–prednisolone, where our data could be useful for molecular signatures of bortezomib-based treatments.

In conclusion, we present a comprehensive single-cell landscape of BM-MNCs in treatment-naïve MM patients receiving bortezomib-based treatments. We unveil the compositional dynamics and interactions between the tumor and TME and the potential biomarkers in predicting bortezomib-based treatment response. Our data may contribute to the current understanding of bortezomib resistance mechanisms in MM. Further functional investigations and discovery of the communication between the tumor and TME will help in establishing novel therapeutic strategies for diagnosing and treating MM refractory to treatment.

## Supplementary information

Supplementary Materials

Supplementary Table 1

Supplementary Table 2

Supplementary Table 3

Supplementary Table 4

Supplementary Table 5

Supplementary Table 6

Supplementary Table 7

Supplementary Table 8

Supplementary Table 9

Supplementary Table 10

Supplementary Table 11

Supplementary Table 12

## Data Availability

Raw sequencing data generated for scRNA-seq have been deposited in the Gene Expression Omnibus under accession number GSE189460.
