# Supplementary material for: Single-cell analysis of multiple myelomas refines the molecular features of bortezomib treatment responsiveness
Source: Exp Mol Med. 2022 Nov 15;54(11):1967–78. doi: 10.1038/s12276-022-00884-z (PMC9723182; doi:10.1038/s12276-022-00884-z)
Supplement: Supplementary file 1 — Supplementary Materials [file 12276_2022_884_MOESM1_ESM.pdf]

Supplementary Materials

Single-cell analysis of multiple myelomas refines the molecular features of bortezomib treatment responsiveness

Seung-Hyun Jung[1,2,†,*], Sung-Soo Park[3,4,†], Ji-Young Lim[3], SeonYong Sohn[1], Na Yung Kim[1,2], Dokyeong Kim[2,5], Sug Hyung Lee[2,6,7,*], Yeun-Jun Chung[2,5,8,*], Chang-Ki Min[3,4,*]

Department of [1]Biochemistry, [2]Biomedicine & Health Sciences, [3]Hematology, Seoul St. Mary's Hematology Hospital, [4]Leukemia Research Institute, [5]Precision Medicine Research Center/IRCGP, [6]Cancer Evolution Research Center, [7]Pathology, [8]Microbiology, College of Medicine, The Catholic University of Korea

[†]These authors contributed equally to this work

***Correspondence to:**

Chang-Ki Min, E-mail: ckmin@catholic.ac.kr

Yeun-Jun Chung, E-mail: yejun@catholic.ac.kr

Sug Hyung Lee, E-mail: suhulee@catholic.ac.kr

Seung-Hyun Jung, E-mail: hyun@catholic.ac.kr

Supplementary Methods

Patients and sample preparation

From 1,353 consecutive patients who diagnosed of symptomatic multiple myeloma, we found 1,247 cases who received bortezomib-based induction treatment (i.e the first treatment) between December, 2014 and January, 2021 at our institution. For establishment the discovery cohort, we obtained 68 cases who treated with bortezomib-melphalan-prednisolone (VMP) and their baseline BM samples. Among these cases and samples, to avoid selection bias, we finally constructed the discovery cohort which consists of 18 consecutive patients and their samples in case VMP was initiated between December, 2018 and September, 2019. The other 50 cases were included in the validation cohort. On the other hand, to enhance power of validation, we additionally found consecutive 40 cases who had baseline BM samples and administered bortezomib-thalidomide-dexamethasone (VTD) as induction treatment between October, 2017 and January, 2021, and these 40 cases also included in the validation cohort (supplementary Fig. 1).

Treatment responses were assessed according to the criteria from the International Myeloma Working Group (IMWG) and classified as complete response (CR), very good partial response (VGPR), partial response (PR), minimal response (MR), stable disease (SD) and progressive disease (PD)[1]. Patients who achieved the best response in CR or VGPR from initiation to the end of treatment were defined as optimal responders, while PR, MR, SD, or PD were defined as suboptimal responders. To identify biomarkers related to the treatment response, bone marrow (BM) aspirate samples were collected at baseline prior to the induction treatment of VMP or VTD. BM aspirates were subjected to a Ficolle-Paque PLUS (GE Healthcare) gradient, and isolated mononuclear cells were cryopreserved in freezing medium (10% DMSO + 90% FBS) before the experiments. Cytogenetic findings and International Staging System (ISS)[2] were taken from the data established at diagnosis. High-risk cytogenetic abnormalities were

defined as the presence of one or more of the following: del(17p), t(4;14), or t(14;16). All specimens from the patients in this study were obtained with appropriate consents in accordance with the declaration of Helsinki. The current study was approved by the institutional review board of the Catholic University of Korea, College of Medicine (KC12SISE0594).

Single-cell RNA sequencing library construction

The single-cell library preparation relied on a commercially available droplet method, the 10x Genomics Chromium System (10x Genomics, Pleasanton, CA), with 10x Genomics Single Cell 3' v3 Reagent kit according to the manufacturer's protocol. In brief, single-cell suspension was counted by hemocytometer (Thermo Fisher Scientific, Waltham, MA) and loaded into a Chromium instrument system targeting 10,000 cells. The cells were then partitioned into gel beads in emulsion (GEMs) in the Chromium instrument, where cell lysis and barcoded reverse transcription of RNA occurred. Complementary DNA (cDNA) was synthesized and amplified for 14 cycles. cDNA clean-up was performed using a SPRIselect Reagent Kit (Beckman Coulter, Brea, CA). 50 ng of the amplified cDNA were used for each sample to construct indexed sequencing libraries. Sequencing libraries were sequenced on an Illumina HiSeq2500 platform. Raw sequencing data generated for scRNA-sequencing have been deposited in Gene Expression Omnibus (GEO) under accession number GSE189460.

Single-cell RNA sequencing data analysis

The sequenced data were processed into expression matrices with the Cell Ranger Single Cell software suite version 3.1.0 (10x Genomics). Raw base-call files from HiSeq2500 sequencer were demultiplexed into library-specific FASTQ files using the Cell Ranger *mkfastq* option. Sequencing reads were mapped to the GRCh38 (version 3.0.0) reference genome

(https://support.10xgenomics.com/single-cell-gene-expression/software/downloads/latest), downloaded from the 10x Genomic. Subsequently, cell barcodes and unique molecular identifiers underwent filtering and correction. Reads associated with the retained barcodes were quantified using the Cell Ranger *count* option and used to build a transcript count table. We obtained 10x Genomics Chromium scRNA-seq data set of healthy bone marrow from 20 individuals (10 males and 10 females with ages ranging from 24 to 84 years old) from GEO accession GSE120221[3].

Bioinformatics processing of the scRNA-sequencing data was performed with the R package Seurat (version 4.0.2)[4]. To exclude low-quality cells in scRNA-sequencing, we filtered cells with an expressed gene count fewer than 2% or greater than 98%. Additionally, we removed cells in which more than 20% of reads corresponded to mitochondrial genes. Data was log-normalized, and highly variable features were identified based on a variance stabilizing transformation (VST) method. All individual datasets were then integrated using the canonical correlation analysis (CCA) method, "FindIntegrationAnchors" and "IntegrateData" functions in Seurat, to remove the sample-specific batch effect. Principal components analysis (PCA) was performed on the integrated datasets. Based on the top 50 principal components (PCs), graph-based clustering was performed using the shared nearest neighbor (SNN) modularity optimization with resolution set to 2, and all cells were classified into 64 clusters. Clustering data was then applied to the uniform manifold approximation and projection (UMAP), allowing the visualization of identified clusters in UMAP plots[5]. Each cell cluster was annotated for their cell type using the SingleR R package[6] (version 1.6.1) and assessment of well-known cell-type-specific markers. Clusters expressed the erythroblast markers (*HBA1*, *HBA2*, and *HBB*) or mesenchymal stem cell markers (*LEPR*, *CXCL12*, and *FSTL1*) were excluded from further analyses.

For sub-clustering analysis, technical differences between two datasets (ours and publically

obtained set) and batches were adjusted using the Harmony algorithm[7]. Cell cycle analysis was performed by using the “CellCycleScoring” function in Seurat. Differentially expressed gene (DEG) analysis was used to identify significant DEGs within each cluster using the logistic regression test for significance and an average log fold-change. We kept only genes with a positive average log fold-change value (greater than 0.25) and an adjusted *P* value lower than 0.05 in the analysis. All single MM cells from each OR and SOR group pooled together for pseudo-bulk DEG analysis of MM cells.

Gene module scores were calculated by taking the mean of the scaled and centered expression value across multiple signature genes using the “AddModuleScore” function in Seurat. Each module score for T cell subsets was generated using the following markers: Naïve module (*CCR7*, *TCF7*, *SELL*, and *LEF1*), Treg module (*FOXP3*, *IL2RA*, *CTLA4*, *RTKN2*, and *DUSP4*), IFN module (*ISG15*, *IFI44L*, *IFI6*, *MX1*, *IFIT3*, *IFI44*, *OAS3*, and *IRF7*), cytotoxic module (*CX3CR1*, *PRF1*, *GZMA*, *GZMB*, *GZMH*, *GZMK*, *GNLY*, *FGFBP2*, *KLRG1*, *FCGR3A*, and *KLRD1*), MAIT module (*KLRB1*, *NCR3*, *CEBPD*, *SLC4A10*, and *LST1*), γδ module (*TRDC*, *TRGC2*, *KLRF1*, *FCGR3A*, *KLRC2*, *KLRD1*, and *CST7*), and dysfunctional module (*PDCD1*, *LAG3*, *HAVCR2*, *TIGIT*, *CD244*, *TOX*, and *CTLA4*). Density plots were generated using the Nebulosa R package (version 1.2.0) with default parameters[8].

Gene ontology and trajectory analysis

Gene ontology (GO) and pathway enrichment analysis were performed using Metascape[9]. The ‘biological process’, ‘cellular components’, ‘molecular function’, and ‘KEGG pathways’ categories were used in this analysis with default parameters (Min Overlap = 3, *P*-Value Cutoff = 0.01, Min Enrichment: 1.5).

Trajectory analyses were performed using the SCORPIUS R package (version 1.0.8)[10]. The method finds the initial path as the shortest path through n number of k-means clusters in the

reduced dimension. We further refined the path iteratively by fitting the path with the principal curves also identifying the optimal linear trajectory. Default parameters of the SCORPIUS package were used with minor changes in that the UMAP cell embedding values were used as input for the 'infer_trajectory' function instead of calculating dimensionality by the SCORPIUS.

Inferred copy number alteration analysis from scRNA-seq

Copy number alterations (CNAs) were inferred from the scRNA-seq data using inferCNV R package v1.9.0 (https://github.com/broadinstitute/inferCNV)[11]. In brief, raw gene expression data of plasma cell subsets were extracted from the Seurat object as a count matrix file. Plasma cells from patients with MM were used as the 'observations', while those from healthy donors were used as the normal 'reference'. To run the inferCNV, following parameters were used: denoise, default hidden markov model (HMM) settings, and a value of 0.1 for "cutoff. To determine the clonal CNAs in each tumor, we utilized the "subcluster" method on the CNAs generated by the HMM. Each CNA was annotated to be either a copy gain or a copy loss.

TaqMan low density gene expression array experiments

To validate the treatment response-related genes using the validation cohort, 44 DEGs (up-regulated: *BCAP31*, *BCL2*, *BST2*, *CCL3*, *CCND1*, *CD320*, *CD53*, *COX5A*, *CXCR4*, *DUSP2*, *EEF1B2*, *EGR1*, *EIF2AK4*, *EIF3M*, *HIST1H1C*, *HSP90AB1*, *IL6ST*, *JUNB*, *LAMP5*, *MS4A1*, *MYC*, *NFKBIA*, *NOP53*, *NPM1*, *PDIA2*, *PIM1*, *PSMA7*, *RACK1*, *RGS1*, *SEC11A*, *SQSTM1*, *SRP9*, *SSR3*, *TNFRSF17*, *TSC22D3*, and *UQCRH*; down-regulated: *ATF5*, *IGF1*, *ITGB7*, *NEB*, *NSD2*, *PPP1R10*, *TIMP2*, and *RHOB*) between optimal and suboptimal responder groups were examined using custom-made TaqMan Low-Density Arrays (TLDA). The 44 genes were initially selected based on the significance levels and previously reported genes strongly related

to MM pathogenesis. In addition, to avoid the selection bias of genes harboring sample-specific expression, genes that expressed more than 20% of our samples (≥4 MM patients) were finally selected for validation. The number of samples expressing for each of the 44 genes had a median of 18 (100%, range: 4-18), suggesting that the selected genes are commonly expressed in most OR and SOR patients. The only exception was *MS4A1* which was selected as a plasma cell dedifferentiation marker gene. Total RNA was purified from $CD138^+$ MM cells. Purified total RNA was then reverse transcribed using High-Capacity RNA-to-cDNA Kit (Thermo Fisher Scientific). TLDA was then performed with the QuantStudio 12K Flex Real-Time PCR System (Thermo Fisher Scientific) using TaqMan Fast Advanced Master Mix (Thermo Fisher Scientific). The expression level of each target gene was calculated using the $2^{-\Delta\Delta Ct}$, where ΔCt is the difference in threshold cycles for the sample in question normalized against the endogenous control gene (18S ribosomal RNA) and expressed relative to the value obtained by the calibrator as described elsewhere[12, 13]. The mean gene expression level of optimal responders was used as a pseudo calibrator. Student's t-test was used to verify the statistical significance.

Cell-cell communication analysis

To examine the cell-to-cell communication between different cell types in MM TME, receptor-ligand interactions were analyzed using CellChat R package (version 1.1.1)[14]. The ligand-receptor pairs in CellChat retrieved from previous studies were divided into four groups: cytokine/chemokine, immune checkpoint, growth factor, and others[14]. Twelve rare cell populations (mono-6, 7, 8, 9, BC-5, 6, 7, MK, HSPC, two proliferating populations, and pre-B) were excluded from the analysis, and to simplify the analysis, some cell subsets were merged as follows; transitional NK (NK-1 and NK-3), normal B cell (BC-1 and BC-4), effector T cell (cytotoxic-4 and cytotoxic-5), helper T cell (helper-1 and helper-2), malignant plasma

cell in optimal responders (MM-OR; PC-4, 5, 8, 14, 16, 17, 19, 23, and 24), malignant plasma cell in suboptimal responders (MM-SOR; PC-1, 2, 3, 6, 7, 9, 10, 11, 12, 20, 21, 22, 25, 26, 27, 28, 29, 30, 31, and 32), and malignant plasma cell in both optimal and suboptimal responders (MM-Mix; PC-13 and 15). Default parameters of the CellChat package were used with minor changes in that the 'population.size = TRUE' was used to consider the effect of cell proportion in each cell group in the probability calculation. To compare the CellChat result between normal BM and MM BM, the 'liftCellChat' function was used. Cell-cell communications if there are only a few cells (< 100) in certain cell groups were filtered out.

Statistical analysis

Fisher's exact test were used for categorical variables. Student's t-test was used for continuous variables. The relationships between proportions of the cell types and treatment responses were evaluated using spearman's rank correlation. Linear discriminant analysis was applied to construct a prediction model for bortezomib-based treatment response. The clinical variable model consists of two significant features (age, absolute lymphocyte count (ALC), and autologous stem cell transplantation (ASCT)) in univariate analysis, and the equation is as follows: Discriminant score = -2.183 + (0.00028×age) + (2.025×ASCT) + (0.866×ALC). The DEG model consists of 24 significant features (*CD53, EIF3M, CD320, BCL2, BST2, COX5A, SEC11A, RACK1, EEF1B2, NPM1, TSC22D3, EIF2AK4, UQCRH, EGR1, JUNB, PSMA7, SRP9, CXCR4, TNFRSF17, CCND1, RGS1, IL6ST,* and *CCL3*) in univariate analysis, and the equation is as follows: Discriminant score = -0.949 + (0.286×*SSR3*) - (0.007×*CD53*) + (0.082×*EIF3M*) + (0.266×*CD320*) + (0.193×*BCL2*) - (0.052×*BST2*) + (0.140×*COX5A*) - (0.574×*SEC11A*) + (0.143×*RACK1*) + (0.105×*EEF1B2*) - (0.290×*NPM1*) + (0.311×*TSC22D3*) - (0.189×*EIF2AK4*) + (0.299×*UQCRH*) + (0.088×*EGR1*) - (0.204×*JUNB*) - (0.416×*PSMA7*) + (0.049×*SRP9*) - (0.039×*CXCR4*) + (0.015×*TNFRSF17*) + (0.006×*CCND1*) - (0.011×*RGS1*) +

(0.012×*IL6ST*) + (0.01×*CCL3*). The combined prediction model consists of the above three clinical features and 24 DEG features, and the equation is as follows: Discriminant score = 1.699 - (0.009×age) - (1.682×ASCT) - (0.677×ALC) + (0.174×*SSR3*) - (0.06×*CD53*) + (0.085×*EIF3M*) + (0.250×*CD320*) + (0.209×*BCL2*) + (0.044×*BST2*) + (0.042×*COX5A*) - (0.493×*SEC11A*) + (0.208×*RACK1*) + (0.057×*EEF1B2*) - (0.263×*NPM1*) + (0.195×*TSC22D3*) - (0.2×*EIF2AK4*) + (0.260×*UQCRH*) - (0.000037×*EGR1*) - (0.053×*JUNB*) - (0.509×*PSMA7*) + (0.131×*SRP9*) - (0.024×*CXCR4*) - (0.046×*TNFRSF17*) + (0.006×*CCND1*) - (0.008×*RGS1*) + (0.084×*IL6ST*) + (0.012×*CCL3*). The receiver operating characteristic (ROC) curve and area under curve (AUC) were used to assess the predictive values of each DEG and prediction model for bortezomib-based treatment. For survival analysis, time-to-event variables were defined as duration from the initiation date of bortezomib-based treatment to the date of disease progression. Patient survival was calculated by Kaplan-Meier method and differences in survival rates between groups were tested with the log-rank test. RNA sequencing data and clinical information for Relating Clinical Outcomes in Multiple Myeloma to Personal Assessment of Genetic Profile (CoMMpass) dataset were obtained from the Multiple Myeloma Research Foundation (https://research.themmrf.org/). Among the 1167 samples available, 522 treatment-naïve primary MM patients who later received bortezomib-based treatment as first-line were used. Statistical analyses were performed using SPSS (version 25, Chicago, IL). GraphPad Prism software (version 8, La Jolla, CA) was used to create graphs. All *P* values less than 0.05 were considered significant in all statistical analyses.

Supplementary Figures

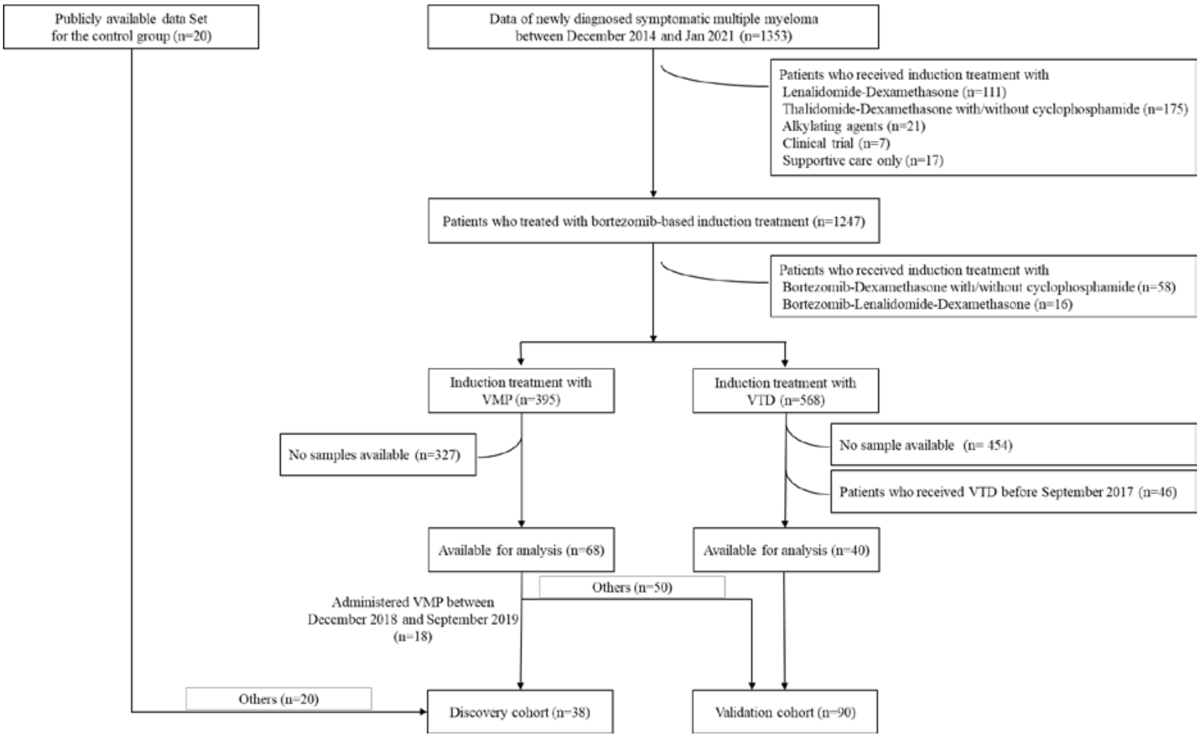

Supplementary Fig. 1. Flow diagram to construct the discovery cohort and validation cohort. The discovery cohort consists of 18 patients who received bortezomib-melphalan-prednisolone from our dataset and 20 healthy donors from publicly available dataset. The validation cohort included 90 patients who received bortezomib-melphalan-prednisolone (n=50) and bortezomib-thalidomide-dexamethasone (n=40).

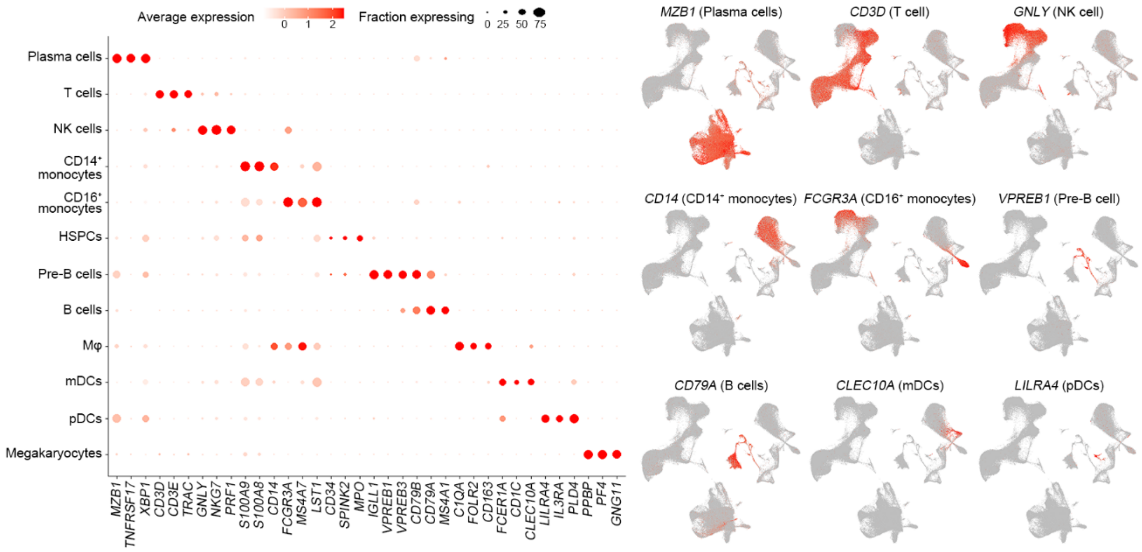

Supplementary Fig. 2. Expression of canonical cell type-defining signature genes. Left: dot plot of cell-type-specific marker genes per cell type. Dot intensity (from white to red) represents the average expression value of all cells per cell type, whereas dot size represents the proportion of cells expressing the genes. Right: feature plots representing the expression levels of selected cell-type-specific marker genes.

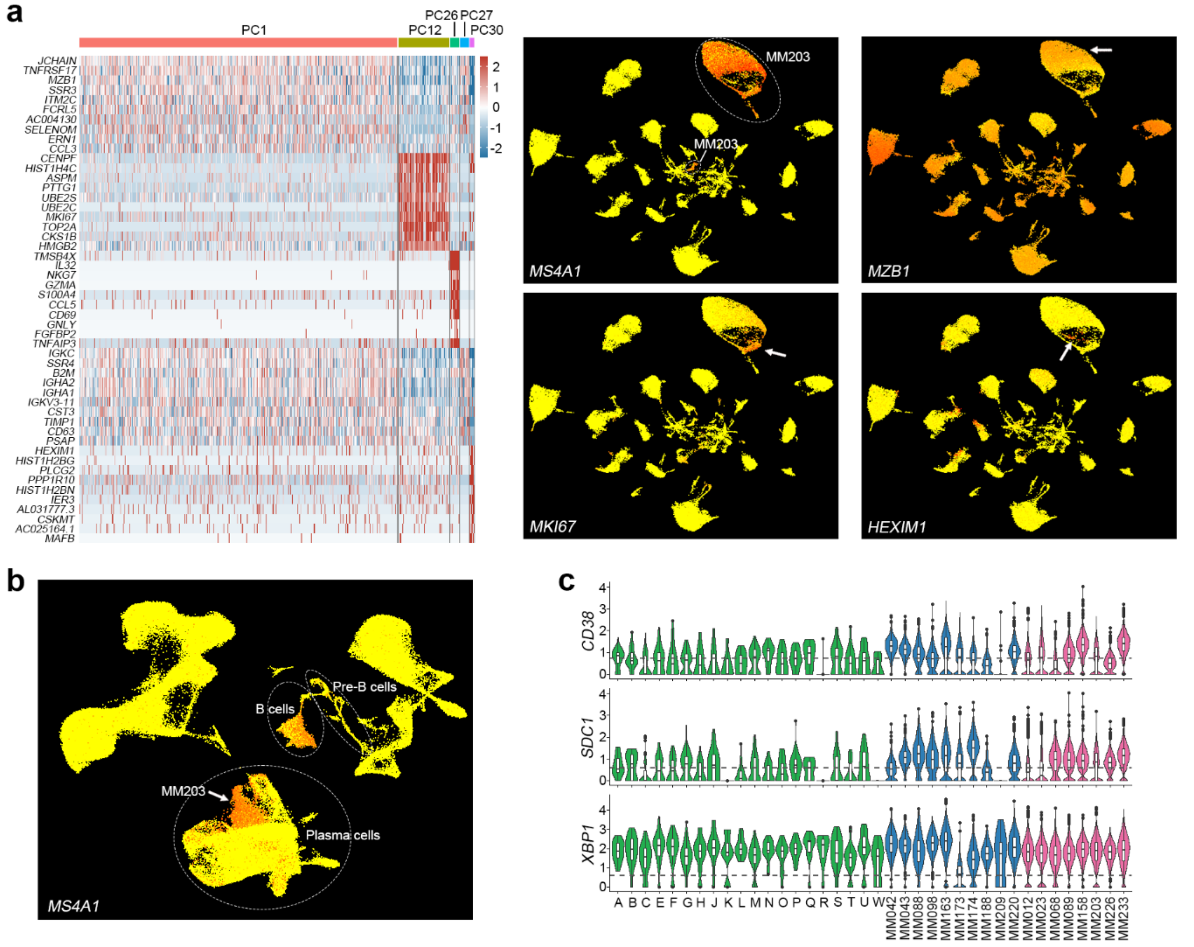

Supplementary Fig. 3. Heterogeneity of malignant plasma cells. (a) Sub-clustering analysis of MM203. Left, the heatmap shows the top 10 most differentially expressed genes in each subset. Blue and red indicate lower and higher expression, respectively. The marker genes are shown on the left. Right, Expression levels of representative patient-specific marker genes are shown. MM203 was characterized by high expression of *MS4A1* (CD20) and harbored five subsets (see Figure 2B-D); PC1 (80.8%; enriched genes: *TNFRSF17* and *MZB1*), PC12 (12.9%; enriched genes: *MKI67* and *TOP2A*), PC26 (2.2%; enriched genes: *NKG7* and *GNLY*), PC27 (2.2%; enriched genes: *SSR4* and *CD63*), and PC30 (1.1%; enriched genes: *HEXIM1* and *IER3*). (b) Expression levels of *MS4A1* in BM-MNCs. The *MS4A1* was expressed in pre-B cells, much more abundant in B cells, but not expressed in plasma cells except MM203. (c) Violin plots present the expression of prototypical plasma cell markers (*CD38*, *SDC1*, and *XBP1*) in

healthy-donors (alphabets ‘A’ to ‘W’) and 18 MM individual patients (‘MM042’ to ‘MM233’). Each violin color-coded according to clinical groups as shown in Fig. 2c. The gray dot line represents the average expression of healthy donors.

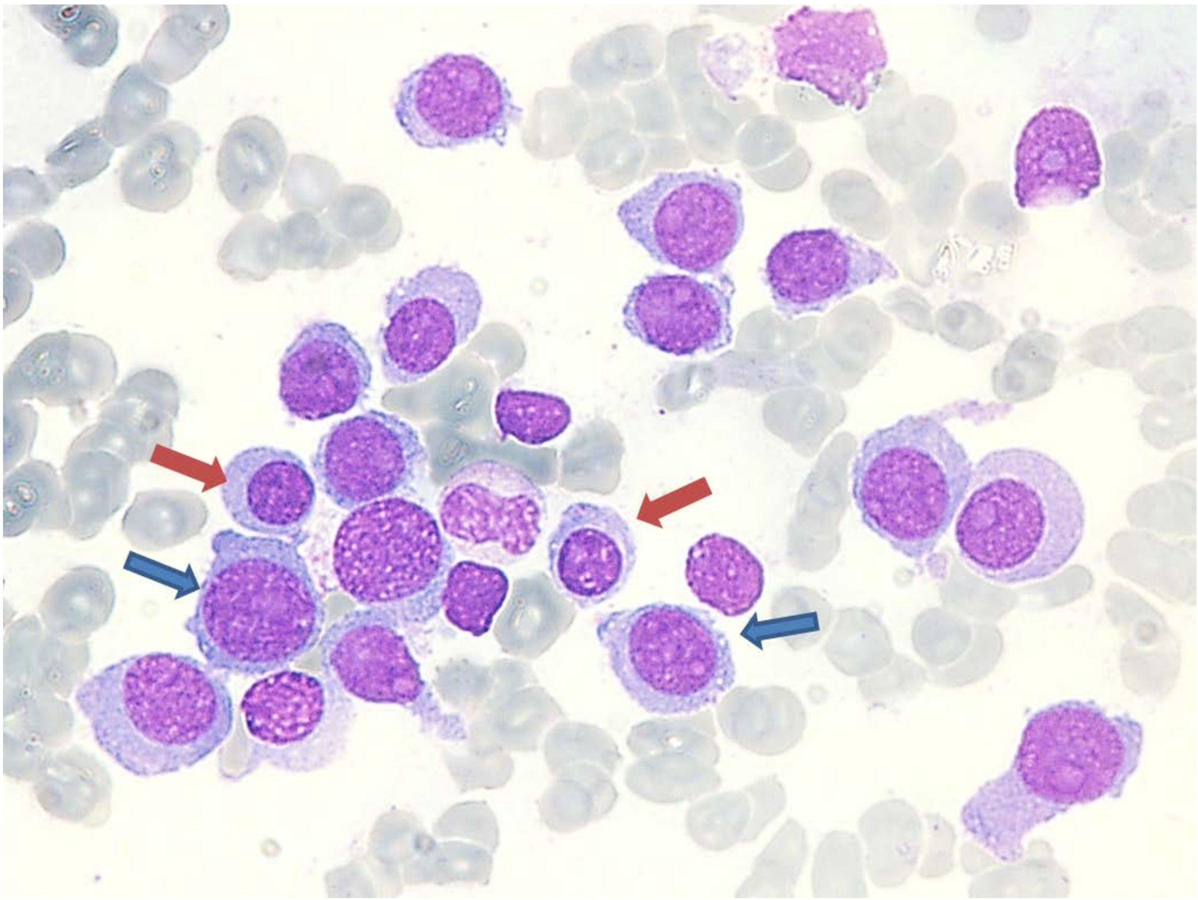

Supplementary Fig. 4. Plasma cell dedifferentiation of MM203. Abundant plasmablasts were founded in the bone marrow. Wright stain x1,000, blue arrow indicates the plasmablast, red arrow indicates the plasma cells.

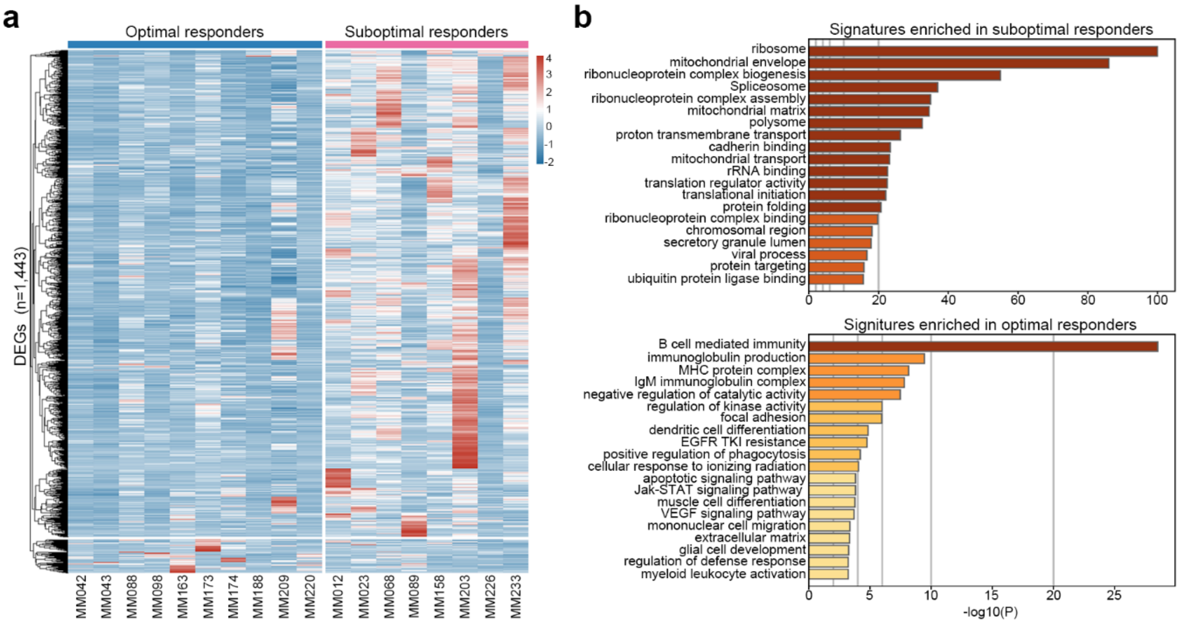

c

Threshold	Sensitivity, %	Specificity, %	Accuracy, %
-0.3135	83.3%	66.7%	76.7%
-0.3045	83.3%	68.5%	77.4%
-0.2800	83.3%	70.4%	78.1%
-0.2310	83.3%	72.2%	78.9%
-0.1810	83.3%	74.1%	79.6%
-0.1570	83.3%	75.9%	80.4%
-0.1515	83.3%	77.8%	81.1%
-0.1020	83.3%	79.6%	81.9%
-0.0360	83.3%	81.5%	82.6%
-0.0135	83.3%	83.3%	83.3%

Threshold	Sensitivity, %	Specificity, %	Accuracy, %
**0.0520**	**83.3%**	**85.2%**	**84.1%**
0.1325	80.6%	85.2%	82.4%
0.1640	77.8%	85.2%	80.7%
0.2290	75.0%	85.2%	79.1%
0.2865	75.0%	87.0%	79.8%
0.3380	75.0%	88.9%	80.6%
0.3985	72.2%	88.9%	78.9%
0.4855	69.4%	90.7%	78.0%
0.5620	66.7%	90.7%	76.3%
0.5735	63.9%	90.7%	74.6%

Supplementary Fig. 5. Prediction model for bortezomib-based treatment response. (a) The heatmap shows the 1,443 DEGs between OR (n = 54) and SOR (n = 36) groups (1,346 upregulated and 97 downregulated in SORs). Red and blue colors indicate upregulated and downregulated genes, respectively. (b) The top 20 statistically-enriched signature terms (Gene Ontology or KEGG pathway) for SORs and ORs. X-axis: log-transformed *P*-values. (c) Sensitivity, specificity, and accuracy by the specified threshold of the combined model. The highest accuracy was observed when the probability score threshold was set at 0.052.

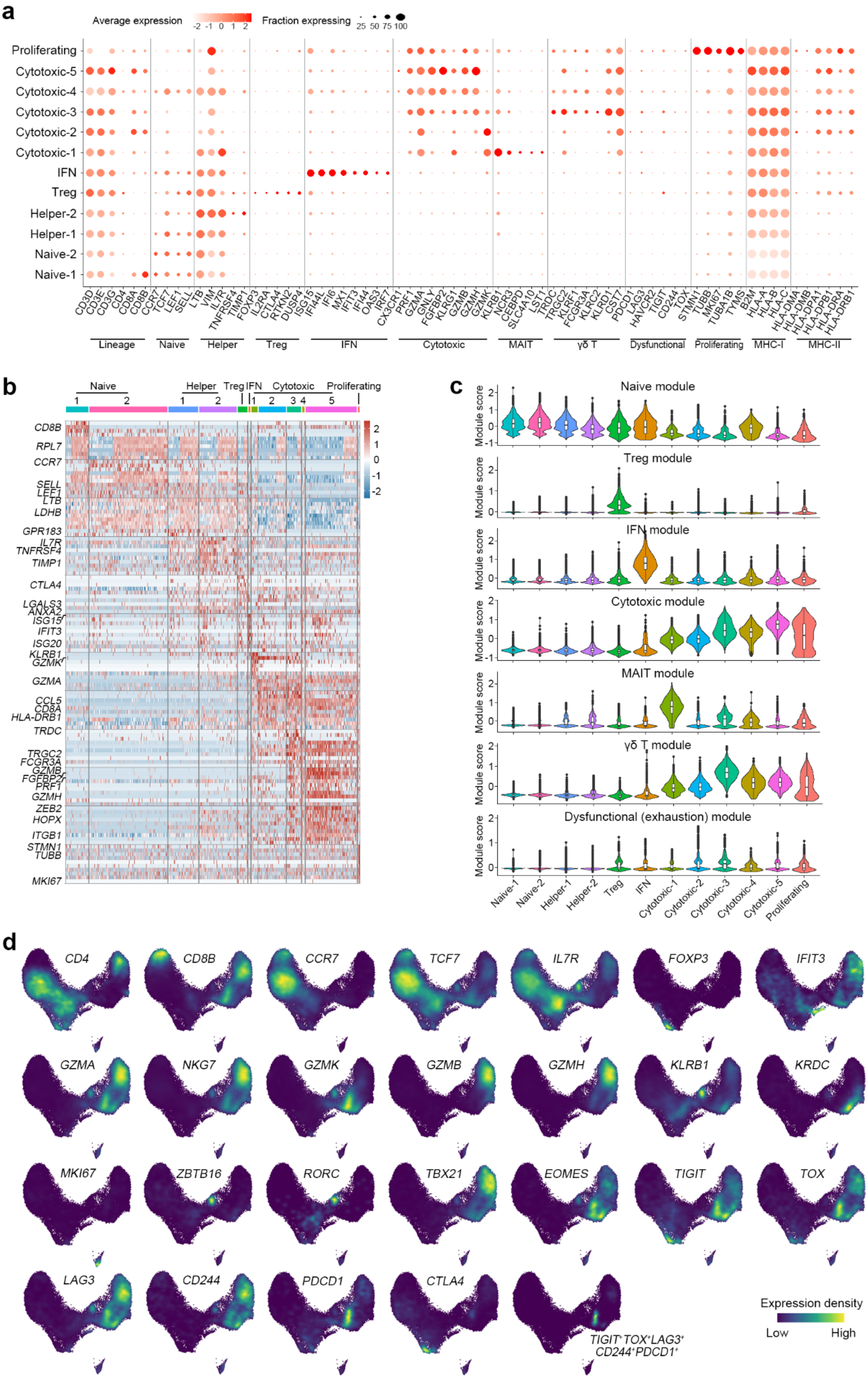
a
Average expression
Fraction expressing
Proliferating
Cytotoxic-5
Cytotoxic-4
Cytotoxic-3
Cytotoxic-2
Cytotoxic-1
IFN
Treg
Helper-2
Helper-1
Naive-2
Naive-1
Lineage
Naive
Helper
Treg
IFN
Cytotoxic
MAIT
γδ T
Dysfunctional
Proliferating
MHC-I
MHC-II
b
Naive
Helper
Treg
IFN
Cytotoxic
Proliferating
c
Naive module
Treg module
IFN module
Cytotoxic module
MAIT module
γδ T module
Dysfunctional (exhaustion) module
Module score
d
CD4
CD8B
CCR7
TCF7
IL7R
FOXP3
IFIT3
GZMA
NKG7
GZMK
GZMB
GZMH
KLRB1
KRDC
MKI67
ZBTB16
RORC
TBX21
EOMES
TIGIT
TOX
LAG3
CD244
PDCD1
CTLA4
TIGIT+TOX+LAG3+
CD244+PDCD1+
Expression density
Low
High

Supplementary Fig. 6. Expression of T cell defining signature genes. (a) dot plot of T cell population-specific marker genes per T subsets. The dot intensity (from white to red) represents the average expression value of all cells per cell subset, and the dot size represents the proportion of cells expressing the genes. Naïve T cell subsets were characterized by the high expression of *CCR7*, *SELL*, and *TCF7*. Of which, the naïve-1 subset highly expressed *CD8B* whereas naïve-2 subset expressed *CD4*, indicating that naïve-1 and naïve-2 are $CD8^+$ and $CD4^+$ naïve T populations, respectively. Helper T cell subsets were characterized by low-level naïve markers and relatively higher expression of *LTB*, *IL7R*, and *TNFRSF4* genes. In addition, helper T cells expressed more MHC-I molecules than naïve T cells. Treg and IFN T populations were characterized by Treg signature genes (*FOXP3*, *IL2RA*, and *CTLA4*) and interferon type I-activated genes (*ISG15*, *IFIT3*, and *IRF7*), respectively[15]. Cytotoxic T cell subsets were characterized by the high expression of cytotoxic markers such as *NKG7*, *PRF1*, and *GZMA* with varying degrees and divided into five subsets. Cytotoxic-1 subset specifically expressed the mucosal-associated invariant T (MAIT) markers such as *KLRB1* (CD161), *CEBPD*, and *SLC4A10* genes but lacked *CD4*/*CD8B* expression and MHC-II molecules expression, indicating the $CD4^-CD8^-$ MAIT (double-negative MAIT)[15, 16]. Cytotoxic-2 subset was *CD8B*$^+$*GZMK*$^+$ memory T population[17]. Cytotoxic-3 subset specifically expressed the *TRDC*, *TRGC1*, and *TRGC2* genes (markers for γδ T) but lacked expression of *CD4* and *CD8B*[18], indicating the $CD4^-CD8^-$ γδ T population. Both cytotoxic-4 and 5 subsets were *GZMB*$^+$*GZMH*$^+$ and had a relatively higher expression level of cytotoxic markers than other cytotoxic subsets (Supplementary Fig. 6d), indicating the terminal effector T population. (b) Heatmap represents the top 10 DEGs in each subset. Blue and red colors indicate lower and higher expression, respectively. The typical markers strongly and specifically associated with each subset are shown on the left. (c) Gene module scores for T cell subsets. Gene module scores were calculated by taking the mean of the scaled and centered expression value across multiple

signature genes. Signature genes for each module are listed in Supplemental Methods. (d) Density plots represent the distribution of selected marker genes for each T cell subset. The expression density of each marker gene was calculated and displayed using the Nebulosa R package. Relatively higher expression of *ZBTB16* and *RORC* (markers for double negative MAIT) than *TBX21* and *EOMES* (markers for $CD8^+$ MAIT) in cytotoxic-1 subset[16]. The *TIGIT* and *TOX* were expressed in both cytotoxic T and Treg, whereas *LAG3* and *CD244* were expressed only in the cytotoxic T population. The *PDCD1* and *CTLA4* were dominantly expressed in memory T and Treg, respectively. Penta-positive dysfunctional T cells ($TIGIT^+TOX^+LAG3^+CD244^+$ $PDCD1^+$) were predominantly observed in the cytotoxic memory T population.

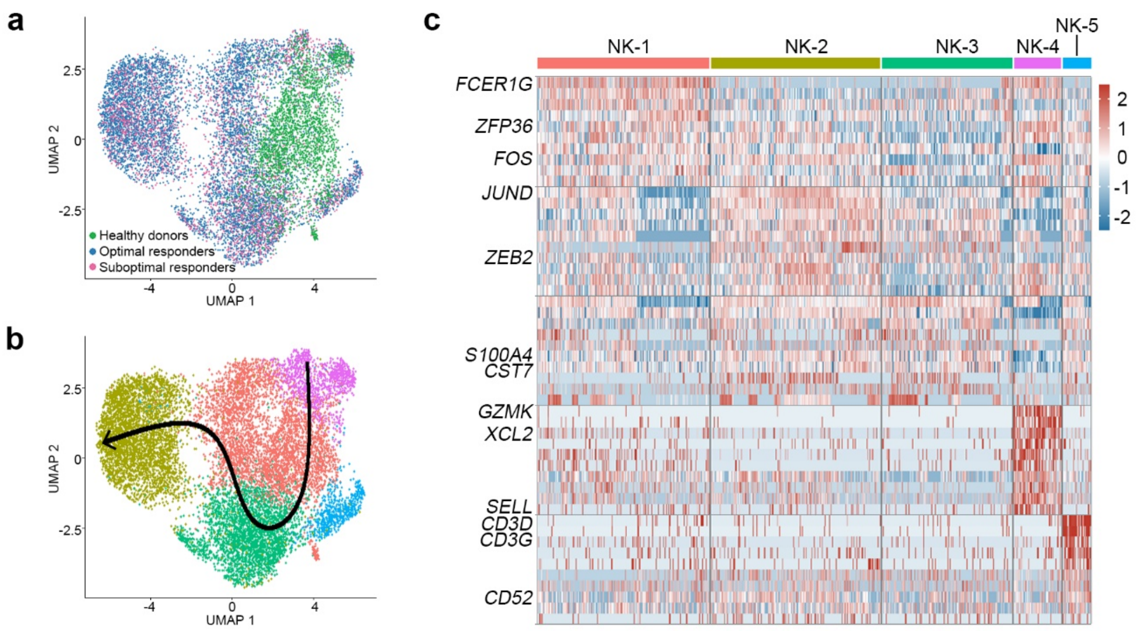

Supplementary Fig. 7. NK cell subsets of MM in scRNA-seq. (a) UMAP plot colored by clinical groups. (b) Pseudo-time trajectory as per pseudo-time algorithm. The trajectory analysis shows a sequential differentiation process of CD56^{bright} (NK-4) → terminally matured NK (NK-1 and NK-3) → NK-2 subset. (c) Heatmap represents the top 10 DEGs in each NK cell subset. Blue and red colors indicate lower and higher expression, respectively. The typical markers strongly and specifically associated with each subset are shown on the left.

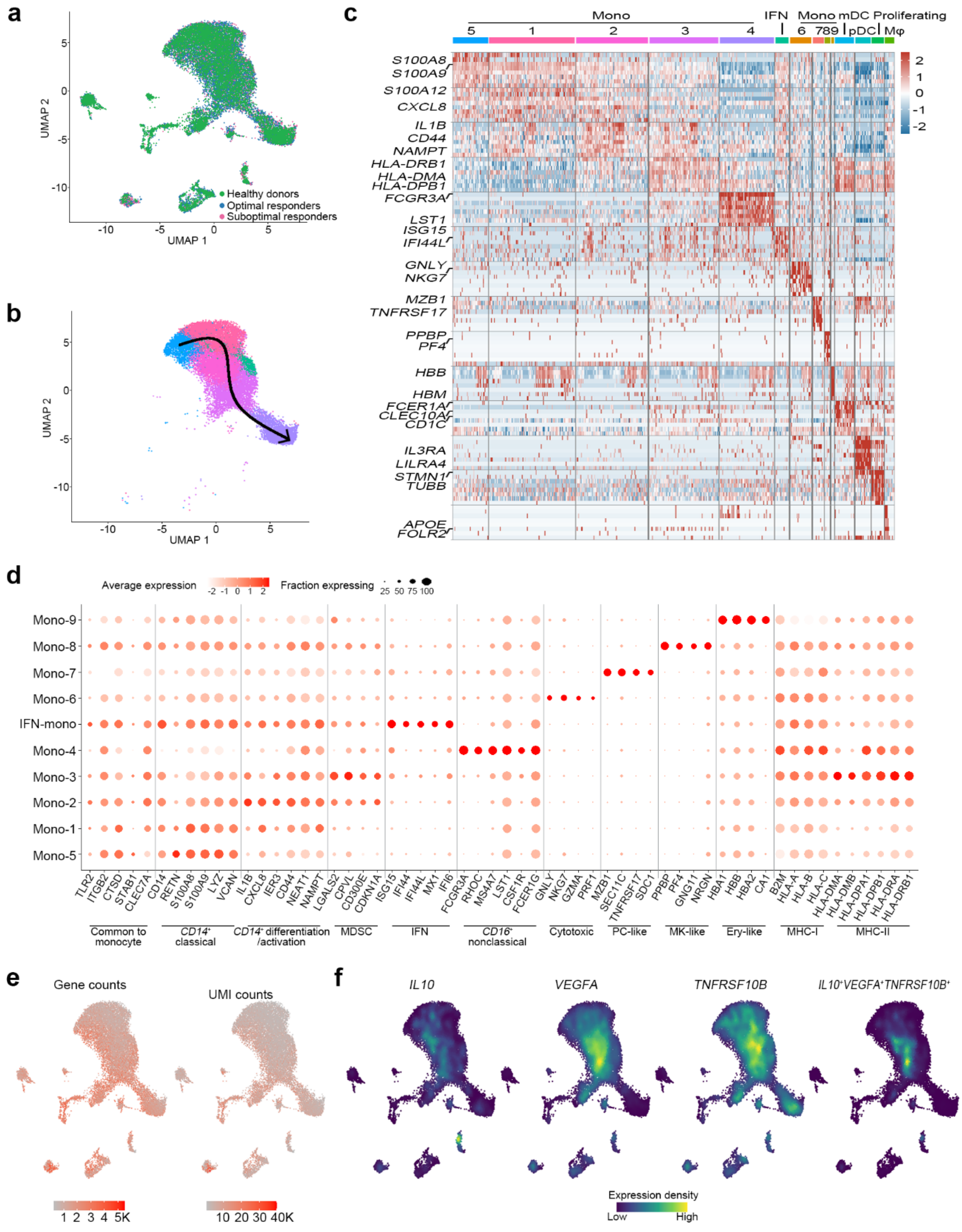

Supplementary Fig. 8. Expression of monocyte defining signature genes. (a) UMAP plot colored by clinical groups. (b) Pseudo-time trajectory as per pseudo-time algorithm. The trajectory analysis shows a sequential differentiation process of classical monocytes (mono-5)

→ transitional monocyte (mono-1) → activated monocyte (mono-2). (c) The heatmap presents the top 10 DEGs in each monocyte/DC/Mφ cell subset. Blue and red colors indicate lower and higher expression, respectively. The typical markers strongly and specifically associated with each subset are shown on the left. (d) Dot plot of monocyte population-specific marker genes per monocyte subsets. The dot intensity (from white to red) represents the average expression value of all cells per cell subset, and the dot size represents the proportion of cells expressing the genes. (e) Gene counts (left) and unique molecular identifier (right) are shown. The mono-6, 7, 8, and 9 subsets distinctively expressed cytotoxic, plasma cell, megakaryocyte, and erythroid gene signatures, respectively. These subsets expressed common genes to all monocytes such as *CTSD*, *CD14*, and *CLEC7A*[19] and did not harbor higher UMI or gene counts than other cell populations, suggesting that possibility of doublet or miss-classification might be unlikely. A previous scRNA-seq study that monocyte subpopulations expressed a cytotoxic gene signature further supports our findings[19]. (f) Density plots represent the distribution of selected marker genes for M-MDSCs. The mono-3 subset was characterized by the high expression of M-MDSCs markers such as *IL10*, *VEGFA*, and *TNFRSF10B*[20, 21]. Although low MHC-II surface expression is the hallmark of MDSC[20], the mono-3 population highly expressed MHC-II molecules. This finding is consistent with a previous study that *CD14*$^+$ monocytes coculture with myeloma cells significantly increased *HLA-DRA* at RNA level but dramatically dropped in the surface expression of HLA-DR and HLA-DP[17]. Therefore, we interpreted the mono-3 subset as M-MDSC-like population. The expression density of each marker gene was calculated and displayed using the Nebulosa R package.

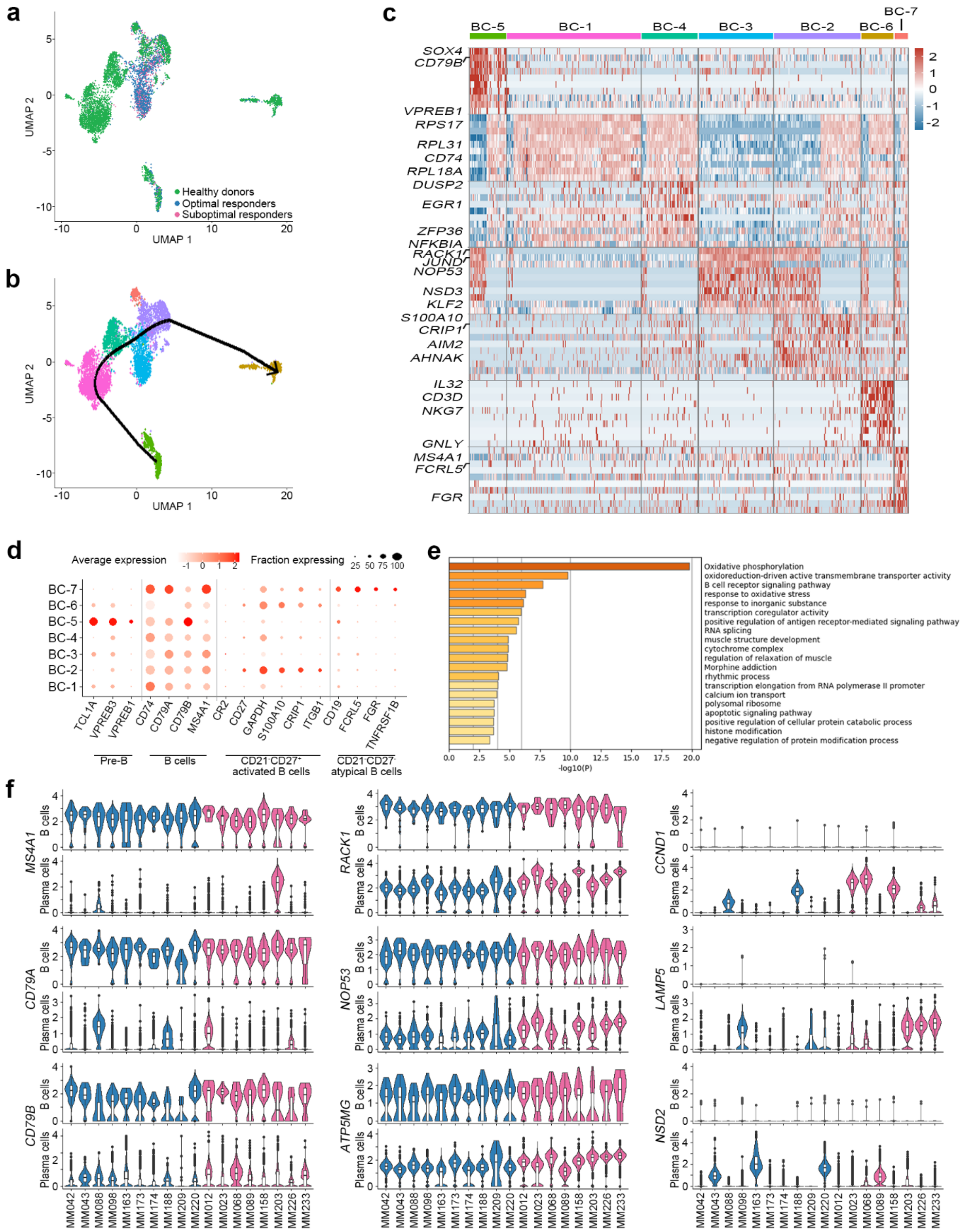

Supplementary Fig. 9. B cell subsets. (a) UMAP plot colored by clinical groups. (b) Pseudo-time trajectory as per the pseudo-time algorithm. (c) The heatmap shows the top 10 most differentially expressed genes in each B cell subset. Blue and red indicate lower and higher

expression, respectively. The typical markers strongly and specifically associated with each subset are shown on the left. (d) Dot plot of B cell population-specific marker genes per B subsets. The dot intensity (from white to red) represents the average expression value of all cells per cell subset, and the dot size represents the proportion of cells expressing the genes. The BC-5, 2, and 7 subsets well corresponded to the previously defined pre-B, $CD21^{-}CD27^{+}$ activated B, and $CD21^{-}CD27^{-}$ atypical B cell populations, respectively[22], whereas the others did not. (e) The top 20 statistically enriched signatures (Gene Ontology or KEGG pathway) for BC-3 subset marker genes (n = 100 genes). The X-axis represents the log-transformed *P*-values. (f) Violin plots present the expression of prototypical B cell markers (*MS4A1*, *CD79A*, and *CD79B*), common genes to MM (*RACK1*, *NOP53*, and *ATP5MG*), and MM driver genes (*CCND1*, *LAMP5*, and *NSD2*) in 18 MM individual patients. Each violin color-coded according to clinical groups as shown in Fig. 2c.

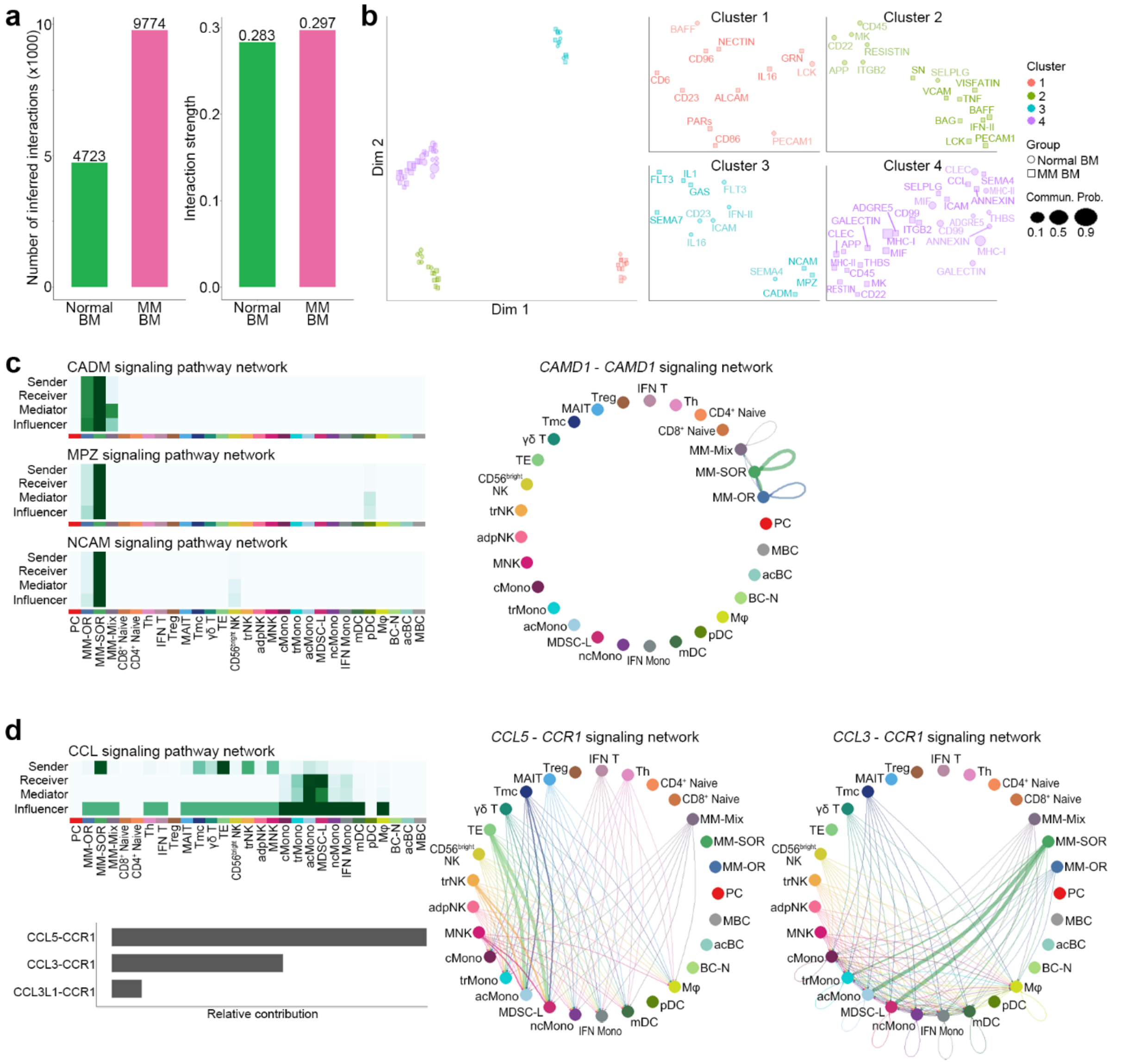

Supplementary Fig. 10. Cell-to-cell communication analysis of tumor and its microenvironment. (a) Comparison of the total number of interactions and interaction strength between normal BM and MM BM. (b) Jointly projecting and clustering signaling pathways from normal BM and MM BM into a shared two-dimensional manifold according to their topological similarity. Circle and square symbols represent the signaling networks from normal BM and MM BM, respectively. Each dot or square represents the communication network of one signaling pathway. Dot or square size is proportional to the total communication probability. Different colors represent different groups of signaling pathways. Some of the same signaling

pathways from normal BM and MM BM, such as FLT3, MHC-I, GALECTIN, and THBS, were grouped together, suggesting that these pathways are essential for both normal and MM. (c) Left, the heatmaps show the relative importance of each cell group based on the computed four network centrality measures of CADM signaling, MPZ signaling, and NCAM signaling networks. The heatmap scale indicates the importance, from white (importance = 0) to dark green (importance = 1). Right, circle plot of CADM signaling network. It shows the inferred autocrine and paracrine signaling to MM BM cell subsets and to normal BM cell subsets, respectively. Edge width represents the communication probability. Edge colors are consistent with the signaling source. (d) Left, the heatmap shows the relative importance of each cell group based on the computed four network centrality measures of CCL signaling networks. The heatmap scale indicates the importance, from white (importance = 0) to dark green (importance = 1). Relative contribution of each ligand-receptor pair to the overall communication network of CCL signaling pathway is shown below. Right, circle plots of *CCL5-CCR1* signaling and *CCL3-CCR1* signaling networks. Edge width represents the communication probability. Edge colors are consistent with the signaling source.

Supplementary References

1. Rajkumar, S.V. *et al.* Consensus recommendations for the uniform reporting of clinical trials: report of the International Myeloma Workshop Consensus Panel 1. *Blood* **117**, 4691-4695 (2011).

2. Greipp, P.R. *et al.* International staging system for multiple myeloma. *J. Clin. Oncol.* **23**, 3412-3420 (2005).

3. Oetjen, K.A. *et al.* Human bone marrow assessment by single-cell RNA sequencing, mass cytometry, and flow cytometry. *JCI Insight* **3**(2018).

4. Stuart, T. *et al.* Comprehensive Integration of Single-Cell Data. *Cell* **177**, 1888-1902 e1821 (2019).

5. Becht, E. *et al.* Dimensionality reduction for visualizing single-cell data using UMAP. *Nat. Biotechnol.* (2018).

6. Aran, D. *et al.* Reference-based analysis of lung single-cell sequencing reveals a transitional profibrotic macrophage. *Nat. Immunol.* **20**, 163-172 (2019).

7. Korsunsky, I. *et al.* Fast, sensitive and accurate integration of single-cell data with Harmony. *Nat. Methods.* **16**, 1289-1296 (2019).

8. Alquicira-Hernandez, J. & Powell, J.E. Nebulosa recovers single cell gene expression signals by kernel density estimation. *Bioinformatics* https://doi.org/10.1093/bioinformatics/btab003 (2021).

9. Zhou, Y. *et al.* Metascape provides a biologist-oriented resource for the analysis of systems-level datasets. *Nat. Commun.* **10**, 1523 (2019).

10. Saelens, W., Cannoodt, R., Todorov, H. & Saeys, Y. A comparison of single-cell trajectory inference methods. *Nat. Biotechnol.* **37**, 547-554 (2019).

11. Tirosh, I. *et al.* Dissecting the multicellular ecosystem of metastatic melanoma by single-cell RNA-seq. *Science* **352**, 189-196 (2016).

12. Jung, S.H. *et al.* Circulating microRNA expressions can predict the outcome of lenalidomide plus low-dose dexamethasone treatment in patients with refractory/relapsed multiple myeloma. *Haematologica* **102**, e456-e459 (2017).

13. Sarasquete, M.E. *et al.* Evaluating gene expression profiling by quantitative polymerase chain reaction to develop a clinically feasible test for outcome prediction in multiple myeloma. *Br. J. Haematol.* **163**, 223-234 (2013).

14. Jun, S. *et al.* Inference and analysis of cell-cell communication using CellChat. *Nat. Commun.* **12**, 1088 (2021).

15. Wang, X. *et al.* Single-Cell RNA-Seq of T Cells in B-ALL Patients Reveals an Exhausted Subset with Remarkable Heterogeneity. *Adv. Sci. (Weinh).* e2101447 (2021).
16. Dias, J. *et al.* The CD4(-)CD8(-) MAIT cell subpopulation is a functionally distinct subset developmentally related to the main CD8(+) MAIT cell pool. *Proc. Natl. Acad. Sci. U S A.* **115**, E11513-E11522 (2018).
17. Zavidij, O. *et al.* Single-cell RNA sequencing reveals compromised immune microenvironment in precursor stages of multiple myeloma. *Nat. Cancer.* **1**, 493-506 (2020).
18. Pizzolato, G. *et al.* Single-cell RNA sequencing unveils the shared and the distinct cytotoxic hallmarks of human TCRVdelta1 and TCRVdelta2 gammadelta T lymphocytes. *Proc. Natl. Acad. Sci. U S A.* **116**, 11906-11915 (2019).
19. Villani, A.C. *et al.* Single-cell RNA-seq reveals new types of human blood dendritic cells, monocytes, and progenitors. *Science* **356** (2017).
20. Veglia, F., Sanseviero, E. & Gabrilovich, D.I. Myeloid-derived suppressor cells in the era of increasing myeloid cell diversity. *Nat. Rev. Immunol.* **21**, 485-498 (2021).
21. Rodriguez-Ubreva, J. *et al.* Prostaglandin E2 Leads to the Acquisition of DNMT3A-Dependent Tolerogenic Functions in Human Myeloid-Derived Suppressor Cells. *Cell. Rep.* **21**, 154-167 (2017).
22. Sutton, H.J. *et al.* Atypical B cells are part of an alternative lineage of B cells that participates in responses to vaccination and infection in humans. *Cell. Rep.* **34**, 108684 (2021).
